# Does Positron Attachment Take Place in Water Solution?

**DOI:** 10.1021/acs.jpcb.4c03627

**Published:** 2024-10-09

**Authors:** Mateus Bergami, Jorge Charry, Andres Reyes, Kaline Coutinho, Márcio T. do N. Varella

**Affiliations:** † Instituto de Física, Universidade de São Paulo, Rua do Matão 1371, CEP 05508-090 São Paulo, SP, Brazil; ‡ Department of Physics and Materials Science, University of Luxembourg, L-1511 Luxembourg City, Luxembourg; § Department of Chemistry, Universidad Nacional de Colombia, Av. Cra. 30 #45-03, 111321 Bogotá, Colombia

## Abstract

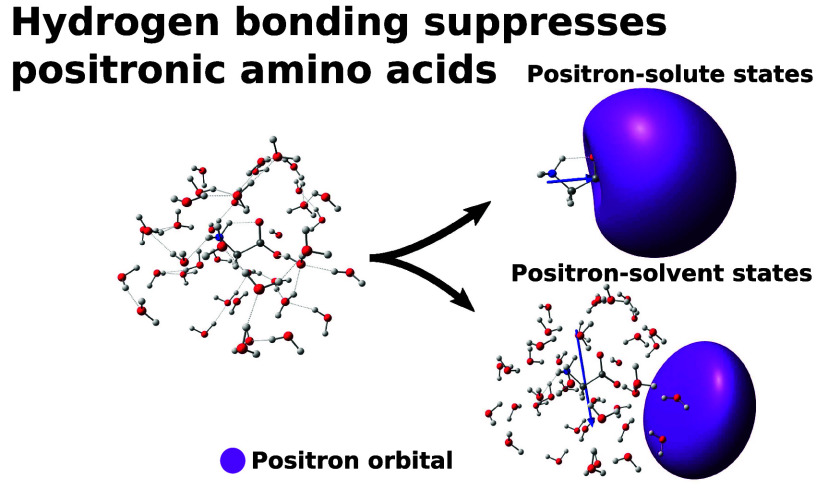

We performed a computational study of positron attachment to hydrated amino acids, namely glycine, alanine, and proline in the zwitterionic form. We combined the sequential quantum mechanics/molecular mechanics (s-QM/MM) method with various levels of any particle molecular orbital (APMO) calculations. Consistent with previous studies, our calculations indicate the formation of energetically stable states for the isolated and microsolvated amino acids, in which the positron localizes around the carboxylate group. However, for the larger clusters, composed of 7 to 40 water molecules, hydrogen bonding between the solute and solvent molecules disfavors positron attachment to the amino acids, giving rise to surface states in which the positron is located around the water–vacuum interface. The analysis of positron binding energies, positronic orbitals, radial probability distributions, and annihilation rates consistently pointed out the change from positron–solute to positron–solvent states. Even with the inclusion of an electrostatic embedding around the aggregates, the positrons did not localize around the solute. Positron attachment to molecules in the gas phase is a well-established fact. The existence of hydrated positronic molecules could also be expected from the analogy with transient anion states, which are believed to participate in radiation damage. Our results indicate that positron attachment to hydrated biomolecules, even to zwitterions with negatively charged carboxylated groups, would not take place. For the larger clusters, in which positron–water interactions are favored, the calculations indicate an unexpectedly large contribution of the core orbitals to the annihilation rates, between 15 and 20%. Finally, we explored correlations between positron binding energies (PBEs) and dipole moments, as well as annihilation rates and PBEs, consistent with previous studies for smaller clusters.

## 1 Introduction

Positron annihilation in condensed matter is a powerful tool for understanding material properties, especially to characterize charged defects,^1^ vacancy,^2^ porous,^3^ and soft matter systems.^4^ The experimental technique commonly employed in these studies is positron annihilation lifetime spectroscopy (PALS), which allows the characterization of material properties.^5^ In addition to materials science applications, positron-electron annihilation enabled the development of Positron Emission Tomography (PET),^6−8^ which is widely used in cancer tumor detection and motivated possible cancer therapy using positrons.^9−11^ Hence, there is a growing interest in understanding the molecular mechanisms of positron interactions with biomolecules.^12−15^

Experimental studies of positron annihilation with molecules in the gas phase are often conducted using low-energy beam techniques.^16−18^ This approach offers valuable information on annihilation rates and positron binding energies, providing evidence of the formation of positron-molecule bound states for a wide range of isolated molecules.^19,20^ The properties of liquids and materials are typically studied with PALS, which searches for signatures of free volumes and chemical environments in the γ radiation produced by pair annihilation.^5,21,22^ PALS measurements also provide positron and positronium (Ps) annihilation rates and lifetimes in condensed matter.

Theoretical methods have been employed to obtain positron binding energies and annihilation rates for atoms,^23−25^ molecules,^26−31^ and clusters with a few solvent molecules.^14,15,32^ Several theoretical studies on isolated atoms and molecules have corroborated the formation of positron-atom and positron-molecule bound states. Achieving a precise description of the binding energies and annihilation rates of positrons presents a significant theoretical challenge, primarily due to computational difficulties in describing electron-positron correlation accurately.^31,33^ In general, electron-positron correlation can only be properly described in small systems, as it requires numerically intensive methods such as Configuration Interaction,^34,35^ Quantum Monte Carlo,^33,36,37^ explicitly correlated Gaussians,^26,38^ generalized Propagator theory^39^ and many-body theory.^31^

Although the description of positron-matter interactions in condensed systems is even more challenging than in the gas phase, we recently proposed^40^ a multicomponent sequential quantum mechanics/molecular mechanics (s-QM/MM) protocol for solvated positronium atoms that provides estimates for binding energies and annihilation rates. In the present work, we apply a similar methodology to investigate the possibility of positron attachment to solvated amino acids. In view of the biomedical applications of positrons (see above), one is led to consider that positronic molecules should exist in aqueous solution, understood as a surrogate for the biological medium, as they do in the gas phase.^19,20^ Small biomolecules are known to attach electrons in the gas phase through a vertical process, thus forming transient negative ions (resonances). These anion states are also believed to be involved in the radiation damage to biomolecules^41,42^ and also in chemo-radiation treatments.^43,44^ The question of positron attachment in the condensed phase, therefore, arises naturally from the analogy with electron attachment.

The choice of amino acids for our study is motivated by previous work on microsolvated positronic complexes of the protein building blocks.^14,15,29^ Specifically, we consider positron binding and annihilation in solvated glycine (Gly), alanine (Ala) and proline (Pro). The molecules are considered in zwitterionic form using the well-established s-QM/MM technique^45^ along with the multicomponent Any Particle Molecular Orbital (APMO) method.^46^ Since we are interested in positron attachment, we first describe the amino acids in thermal equilibrium with solvent. Statistically uncorrelated solute–solvent configurations are then selected for the QM/MM calculations, in which a positron is added to the QM region considering the attachment as a vertical process from the analogy with the electronic case. In addition to this analogy, we consider positron attachment a vertical process also based on the conclusions of Kita et al. about the positron attachment to microsolvated halides.^47^ This study analyzed the effects of geometry relaxation, demonstrating negligible differences in the structure and PBEs of the clusters. Our results suggest that positron attachment would compete with hydrogen bonding, since both the antiparticle and the positively charged hydrogen atoms are attracted to the negatively charged carboxylate groups. This causes the positron to prefer to bind to the solvent rather than to the amino acids under bulk conditions.

This paper is organized as follows. Section 2 describes the sequential QM/MM method and summarizes the Any Particle Molecular Orbital quantum techniques. Section 3 discusses the results of the liquid structure and the effect of the solvent on the positron binding energy and annihilation rates considering the sequential QM/MM procedure. Section 4 presents the conclusions and perspectives.

## 2 Methods

As outlined above, our solvation model is based on the s-QM/MM protocol, which involves classical Monte Carlo (MC) simulations followed by quantum calculations exploring the statistically uncorrelated solute–solvent configurations. In the following, we describe the essential aspects of the s-QM/MM procedure and the APMO employed in the quantum calculations.

### 2.1 S-QM/MM Protocol

In the first s-QM/MM^45^ step, we performed classical MC simulations with the Metropolis algorithm implemented in the DICE software.^48^ We used a simulation box containing 1000 water molecules and one amino acid. The geometries of the molecules were kept rigid, so that only the translation-rotation configuration space was sampled during the simulations. We employed the isothermal–isobaric (*NpT*) ensemble at *T* = 298.15 K and *p* = 1 atm, as well as the standard numerical procedures described in the Supporting Information (SI). The solvent molecules are described with the geometry of the SPC/E force field.^49^ The geometries of the solute molecules in the zwitterionic form were optimized with the MP2/aug-cc-pVDZ level using the Gaussian09 package.^50^ Since the zwitterionic amino acids are unstable in the gas phase, solvation effects were considered with the polarizable continuum model (PCM).^51^ Under physiological conditions, most biomolecules, such as amino acids, peptides, and proteins, occur predominantly in the zwitterionic form,^52−54^ which is considered in the present study. The deprotonation of the carboxyl groups produces a full negative charge in the zwitterion and thus strongly attracts the positron.

Thermal equilibrium was achieved in the MC procedure after 3 × 10^8^ steps. Subsequently, we performed 6 × 10^8^ steps in the production stage. From the latter step, we selected 200 statistically uncorrelated configurations for the liquid systems composed of the amino acid and 1000 water molecules, based on the energy autocorrelation function. The calculated average density (⟨ρ⟩ = 0.99 ± 0.00 g/cm^3^) is consistent with the SPC/E and OPLS-AA^49,55−57^ force fields. The final s-QM/MM step consists in performing APMO calculations for each configuration in the statistically uncorrelated ensemble. The averages of quantum properties reported in this work considered 100 uncorrelated configurations, which were sufficient to obtain converged results.

### 2.2 Any Particle Molecular Orbital Method

In quantum calculations, atomic nuclei were treated as classical point charges under the Born–Oppenheimer approximation, while electrons and positrons were described as quantum particles with the multicomponent APMO approach implemented in the LOWDIN package.^46,58^ Initially, the wave functions were computed at the APMO/Hartree–Fock (APMO/HF) method. Some level of correlation was considered in the positron binding energies (PBEs), which were computed with the generalized APMO/second-order propagator (APMO/P2) approach, accounting for electron–electron and electron-positron single and double excitations. The annihilation rates were calculated from the APMO/HF level wave function, although employing enhancement factors. The HF-level binding energies, indicated as PBE_HF_, were obtained from the difference between the APMO/HF energies of the isolated system (X) and its corresponding positronic complex (Xe^+^),
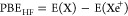
1while Koopmans’ theorem (KT) estimates were obtained from the positronic singly occupied molecular orbital (SOMO),
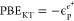
2The PBE_KT_ energies can be improved by including relaxation and correlation corrections via the APMO/P2 self-energy term, ∑_pp_^e^+^^(ω_p_^*e*^+^^), such that
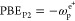
3where ω_p_^*e*^+^^ is the optimized positronic SOMO energy obtained by solving equation
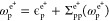
4iteratively with
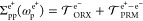
5In the expression above,  is the pair removal correlation term associated with the e-p correlation, and the  term describes electron relaxation upon positron attachment.^27,39^

The APMO/HF wave functions readily provide spin-averaged two-photon annihilation rates (Γ_HF_) for the hydrated amino acids,
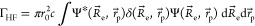
6where *r*_0_ is the classical electron radius, *c* is the speed of light, and Ψ is the APMO/HF wave function. The positron position is indicated as *r⃗*_p_, while the electronic coordinates are collectively indicated as *R⃗*_e_. The APMO/HF annihilation rate can be expressed in terms of the overlaps between electronic and positronic densities
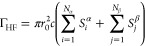
7where *S*_*i*_^α^ = ∫ d**r**|ϕ_α_^*i*^(**r**)|^2^ |φ(**r**)|^2^ is the overlap between the density of the *i*th electronic orbital with α spin (ϕ_α_^*i*^) and the density of the occupied positronic orbital (φ). Similarly, the overlap between the β-spin density and the positron density is represented by *S*_*i*_^β^. The overlaps and radial probability densities were calculated on a numerical grid using the Becke’s multicenter algorithm^59^ implemented in the Multiwfn package.^60^ Since the HF method is known to underestimate the annihilation rates significantly, we employed the enhancement factors (γ_*i*_) proposed by Green and Gribakin,^61^
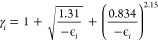
8where ϵ_*i*_ are the electronic orbital energies. The corrected HF-level annihilation rates are given by
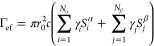
9

## 3 Results and Discussion

### 3.1 Liquid Structure

**Figure 1:**
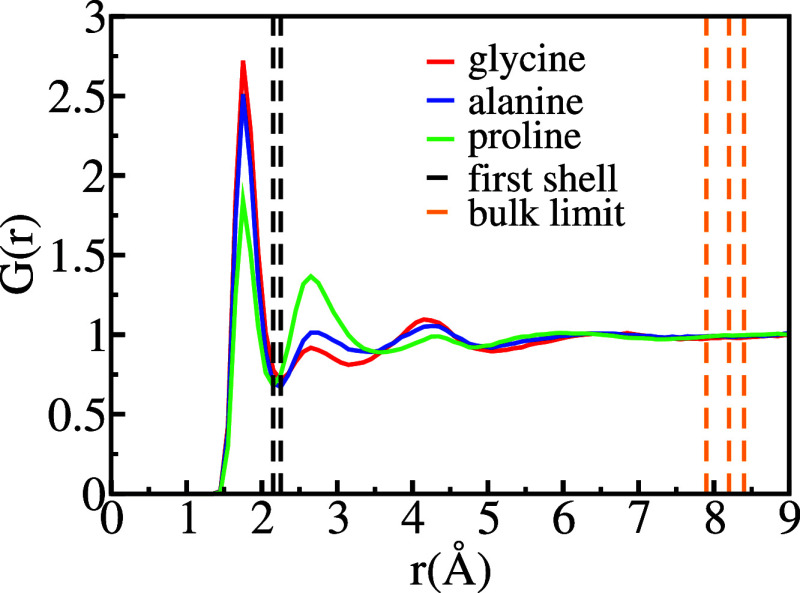
Minimum distance distribution function, *G*(*r*), employing parallelogramic normalization with dimensions 6 Å × 6 Å × 3 Å. The dashed lines define the first solvation shell (black) and bulk limit (orange) in which *G*(*r*) = 1.

The liquid structure obtained in classical simulations plays a pivotal role in defining the quantum (QM) region to be considered in the APMO calculations. Typically, the first hydration shell is the most critical region for characterizing the properties of the solute molecule. It is defined by the first local minimum of the minimum distance distribution function (MDDF)^62^ as depicted in Figure 1. The dashed black lines indicate the first local minima for glycine (2.25 Å), alanine (2.25 Å), and proline (2.15 Å). Integration of the MDDF to the first minima reveals seven water molecules in the first solvation shell for glycine and alanine, whereas six water molecules for proline. These findings are consistent with other studies that predict a first solvation shell consisting of 6–10 molecules for those amino acids in water.^63^ The second solvation shell is also evident, and the bulk behavior, corresponding to MDDF(*r*) = 1, is achieved beyond 8.4, 8.2, and 7.9 Å, respectively for glycine, alanine, and proline, as indicated by the dashed orange lines. Integration of the MDDFs reveals the presence of approximately 150 solvent molecules up to the bulk limit in all cases. Furthermore, the MDDFs also indicate the existence of solute cavities with a radii around 1.55 Å for the three amino acids.

Another essential aspect of the liquid structure is the presence of H-bonds between the solute and the solvent. The average number of H-bonds, calculated for the sets of uncorrelated configurations, was 7.3, 7.4, and 6.6 for glycine, alanine, and proline, respectively, as detailed in the Supporting Information (SI).

### 3.2 Positron Binding Energies

Accurate representation of the positron density heavily relies on selecting appropriate centers for the Gaussian basis set. Ideally, it would require expansion over all atomic centers. However, the computational cost would be large and even prohibitive. It is well-known that positrons tend to localize around negatively charged regions on polar molecules, as discussed in previous studies.^27^ We conducted exploratory studies using an ensemble of liquid configurations encompassing the solute and the first solvation shell, along with expansion centers on different atoms. The positronic basis sets were generated with the even-tempered procedure described elsewhere.^39^

Our exploratory studies indicated that a proper balance between precision and computational cost is achieved with positronic expansion centers on the oxygen atoms of the carboxyl group. The even-tempered positronic basis sets are combined with the electronic basis set 6-31G++(d,p) on the atomic centers of the amino acids, as well as 6-31G+(d,p) basis on the atomic centers of water molecules (see SI). Consequently, all quantum calculations employed two positronic expansion centers on the oxygen atoms of the carboxyl group of the amino acid. The basis set combinations are indicated as 7s7p7*d*/6-31G++(d,p)/6-31G+(d,p) for the positron/amino acid/water system.

The solvent effects were investigated using distinct QM regions, differing in the number of water molecules. Recalling that 6–7 water molecules form the first solvation shell, we considered models with 1, 2, 3, 4, 5, 6, 7, 14, 30, and 40 solvent molecules in the QM region, in addition to the amino acids and the positron. Because the computational effort scales rapidly with respect to the size of the QM region, we did not perform ensemble averages for the models with 40 quantum solvent molecules. For those larger models, we computed results for a single solute–solvent configuration, which was chosen randomly from the uncorrelated ones. The results, including vertical PBEs obtained at the APMO/HF (PBE_HF_) level, Koopmans’ theorem (PBE_KT_), and second-order propagators APMO/P2 (PBE_P2_) are presented in Table 1 for clusters containing 3, 7, 14, and 30 solvent molecules. Similar results for other cluster sizes are available as SI.

**Table 1:** Positron Binding Energies Obtained from APMO/HF (PBE_HF_), APMO/Koopmans’ Theorem (PBE_KT_), and APMO/P2 (PBE_P2_) Calculations for Glycine (Gly), Alanine (Ala), and Proline (Pro) in the Zwitterionic Forms^a^

species	PBE_HF_(meV)	PBE_KT_(meV)	PBE_P2_(meV)	⟨μ⟩ (Debye)	*R*_1_^2^
Gly	507.5	585.3	750.8	11.71	
Gly(H_2_O)_3_	402.5 ± 15.2	462.3 ± 17.4	567.4 ± 18.6	13.58 ± 0.24	0.36
Gly(H_2_O)_7_	393.9 ± 15.9	447.5 ± 18.1	522.5 ± 19.0	15.91 ± 0.31	0.60
Gly(H_2_O)_14_	420.0 ± 22.1	476.8 ± 25.0	533.2 ± 25.9	18.07 ± 0.46	0.63
Gly(H_2_O)_30_	421.4 ± 27.2	467.5 ± 29.7		23.68 ± 0.92	0.73
Ala	498.9	585.6	754.7	11.35	
Ala(H_2_O)_3_	392.8 ± 14.5	458.4 ± 17.4	573.8 ± 19.1	12.85 ± 0.21	0.18
Ala(H_2_O)_7_	333.6 ± 14.2	385.4 ± 16.6	463.7 ± 18.0	14.81 ± 0.32	0.32
Ala(H_2_O)_14_	311.3 ± 18.7	363.6 ± 22.1	423.0 ± 23.4	15.19 ± 0.47	0.51
Ala(H_2_O)_30_	276.8 ± 20.9	312.2 ± 23.7		18.49 ± 0.72	0.42
Pro	577.8	684.4	866.1	12.11	
Pro(H_2_O)_3_	443.5 ± 15.3	519.0 ± 18.3	626.3 ± 19.6	14.34 ± 0.23	0.22
Pro(H_2_O)_7_	417.4 ± 17.2	482.6 ± 20.3	560.2 ± 21.7	16.13 ± 0.34	0.30
Pro(H_2_O)_14_	428.3 ± 25.9	493.7 ± 30.0	556.2 ± 31.3	17.76 ± 0.52	0.50
Pro(H_2_O)_30_	390.4 ± 28.5	434.7 ± 31.4		22.60 ± 0.96	0.71

a The HF-level dipole moments (⟨μ⟩) and correlation coefficients (*R*_1_^2^) for linear regressions between PBE_HF_ and ⟨μ⟩ are also shown. Calculations were performed for isolated and solvated amino acids with the 7s7p7*d*/6-31G++(d,p)/6-31G+(d,p) basis set combination. All values correspond to the averages over all 100 configurations. The results are presented with the respective standard error of the average, except for the isolated amino acid results.

The PBE_HF_ values obtained for the isolated amino acids show some variation compared to previous studies. Charry et al. obtained 562.6, 531.7, and 573.8 meV for glycine, alanine, and proline, respectively, while Nummela et al. predicted 313.9 meV for glycine.^14,27^ These differences can be attributed to variations in the geometries and basis sets used in previous studies. Furthermore, our PBE_KT_ results are higher than those obtained by computing the differences between the HF energies of the positronic and purely electronic systems. The higher binding energies can be attributed to the partial cancellation between the missing relaxation and the correlation energies, which exhibit opposite signs in the PBE_KT_ calculations.^27^ Apart from the APMO/HF level calculations of PBE for isolated amino acids, the positron-electron correlation effects included in the APMO/P2 results (PBE_P2_) significantly improve the PBE_HF_ and PBE_KT_ estimates. The PBE_P2_ results are obtained via the self-energy correction (Σ_pp_^e^+^^), which can be decomposed into the  component associated with the e-p correlation and  associated with electronic orbital relaxation, according to eq 5. In Table 2, we present the  and  corrections for the isolated and solvated amino acids. These results indicate that electronic relaxation tends to reduce the binding energy, whereas the e-p correlation tends to increase it. The net result of these two contributions to the self-energy leads to an overall increase in PBE_KT_, in consistency with a previous study.^27^ These conclusions are further supported by the results obtained when amino acids are considered in the presence of explicit (QM) water molecules.

**Table 2:** Decomposition of the APMO/P2 Positron Binding Energy Into the Koopmans’ (PBE_KT_), Relaxation (), and Correlation () Contributions for Glycine (Gly), Alanine (Ala), and Proline (Pro) in the Zwitterionic Forms^a^

species	PBE_KT_(meV)		
Gly	507.5	–65.1	230.7
Gly(H_2_O)_3_	402.5 ± 15.2	–51.1 ± 2.4	156.2 ± 6.1
Gly(H_2_O)_7_	393.9 ± 15.9	–46.5 ± 2.7	121.5 ± 5.1
Gly(H_2_O)_14_	420.0 ± 22.1	–49.6 ± 3.5	106.0 ± 5.9
Ala	498.9	–73.8	242.9
Ala(H_2_O)_3_	392.8 ± 14.5	–57.1 ± 3.1	172.5 ± 7.2
Ala(H_2_O)_7_	333.6 ± 14.2	–45.3 ± 3.0	123.6 ± 6.2
Ala(H_2_O)_14_	311.3 ± 18.7	–46.3 ± 3.9	105.7 ± 6.7
Pro	577.8	–88.4	270.2
Pro(H_2_O)_3_	443.5 ± 15.3	–64.2 ± 3.2	171.5 ± 6.3
Pro(H_2_O)_7_	417.4 ± 17.2	–56.1 ± 3.7	133.7 ± 6.7
Pro(H_2_O)_14_	428.3 ± 25.9	–57.3 ± 4.9	119.8 ± 7.3

a Calculations were performed for isolated and solvated amino acids with the 7s7p7*d*/6-31G++(d,p)/6-31G+(d,p) basis set combination. All results are presented with the corresponding standard error of the average, except for isolated amino acid results.

For all systems and levels of theory presented in Table 1, the average PBEs show reductions with increasing cluster size up to the first solvation shell. Our analysis of solute-water hydrogen bonds indicated a predominance of water molecules from the first solvation shell involved in these hydrogen bonds. Therefore, this reduction of PBEs for QM regions up to the first layer indicates a strong influence of solvent molecules involved in solute-water hydrogen bonds. The positively charged hydrogens tend to compete with the positron to interact with the solute’s oxygen pair, reducing PBEs at all calculation levels. On the other hand, for large clusters with 14 and 30 water molecules, we did not observe trends for PBEs. Gas-phase experiments indicate that positrons form nearly bound states with isolated water molecules (shallow virtual states).^20^ HF-level PBE estimates below 40 meV were reported for hydrogen-bonded binary clusters^32^ (although CI calculations indicated binding energies up to 140 meV). Our results for larger clusters indicate binding energies around 300 to 400 meV, indicating that even with the solvent effect, the positron is still under the influence of negative local charge from the amino acids with sizable PBEs.

The self-energy correction, given by the difference between PBE_P2_ and PBE_KT_ in Table 1, is reduced from ≈170 meV in the isolated amino acids to ≈60 meV in the larger clusters. The decomposition shown in Table 2 indicates that the smaller self-energy values arise mainly from the decrease in the correlation contribution () with respect to the cluster size (see the SI for other cluster sizes). Although the observed trend suggests that the self-energy correction would become less significant for larger aggregates, they still provide sizable corrections of 56.4, 59.4, and 62.5 meV for Gly(H_2_O)_14_, Ala(H_2_O)_14_ and Pro(H_2_O)_14_, respectively. A similar conclusion was reached for aggregates containing 22 water molecules and the positronium atom,^40^ where a difference of 80 meV was found between the energies PBE_KT_ and PBE_P2_. In general, the results for the larger clusters, containing 14 water molecules, consistently indicate a decrease in the self-energies related to the inclusion of solvent molecules.

**Figure 2:**
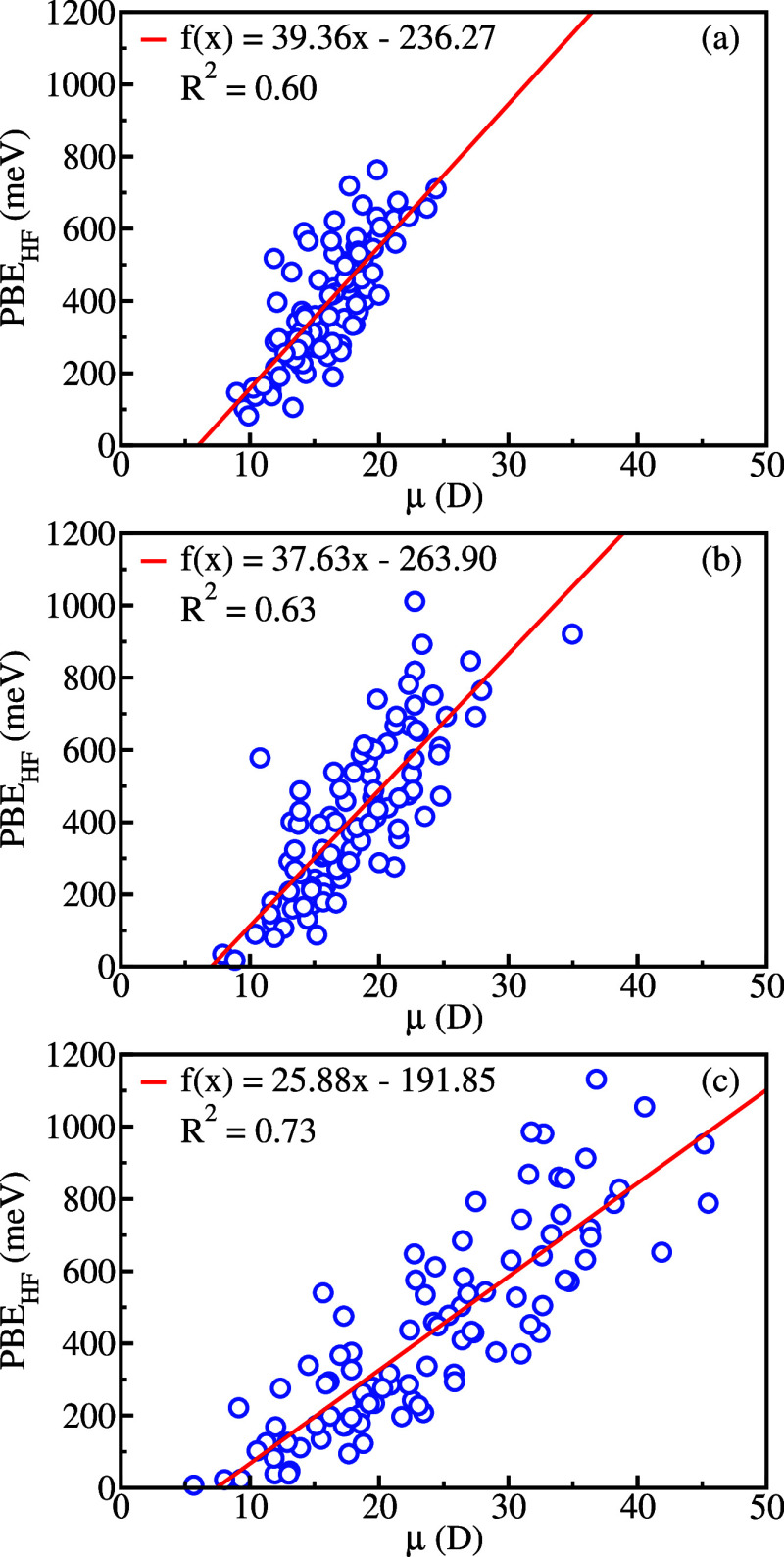
Linear regressions between the HF-level positron binding energies (PBE_HF_) and dipole moments (μ) for glycine clusters: (a) Gly(H_2_O)_7_; (b) Gly(H_2_O)_14_; (c) Gly(H_2_O)_30_. The linear regressions were performed for the sets of uncorrelated liquid configurations.

We further investigated the relation between dipole moment magnitudes and PBEs addressed in previous studies. According to the static dipole model, molecules with supercritical dipole moments (μ > 1.625D) should bind a positron,^64^ although the empirical critical value of μ ≈ 2.5D has been suggested.^65^ Linear regressions of experimental data indicated that PBEs correlate with dipole moments and polarizabilities for gas-phase molecules.^66^ While similar trends were pointed out by theoretical studies of positron attachment to isolated molecules,^27,29,67^ Nummela et al.^14^ found a weak correlation between PBEs and dipole moments for microsolvated biomolecules. Table 1 also shows ensemble averaged HF-level dipole moments, denoted as ⟨μ⟩, which monotonically increase with the cluster size. Since we consider zwitterionic amino acids, the dipole moments largely exceed the critical values. We performed linear regressions of the PBEs to the dipole moments for the sets of statistically uncorrelated configurations. The correlation coefficients *R*_1_^2^ for PBE_HF_ also presented in Table 1, tend to increase with the cluster size, although not monotonically for alanine-water clusters. The linear regressions are shown in Figure 2 for glycine clusters with different numbers of water molecules. The correlation coefficients are rather small for clusters containing up to three solvent molecules (see SI for other cluster sizes), in agreement with ref^14^ However, the coefficient *R*_1_^2^ increases with the size of the glycine-water clusters, although the trend was not as clear for the alanine clusters (see SI). For aggregates with 30 water molecules, we obtained *R*_1_^2^ = 0.73, 0.43, and 0.71, respectively, for glycine, alanine, and proline. The weaker correlation obtained for alanine can be understood, at least in part, from the less polar aggregates, compared to the glycine and proline counterparts (see Table 1). We draw similar conclusions from linear regressions of PBE_P2_ and PBE_KT_ concerning HF-level dipole moments which resulted in the smaller correlation coefficients presented in SI (Tables S5, S6, and S7). In the PBE_HF_ level, the dipole interaction would be expected to drive the binding process due to the lack of *e*-*p* correlation. As the latter effect is partially included in the PBE_P2_, the PBEs become less correlated with the dipole moment magnitudes in these cases. The higher binding energies observed for PBE_KT_ are attributed to the partial cancellation between the missing relaxation and the correlation energies, which also resulted in the smaller correlation coefficients indicating less importance of dipole moment in the binding process.

Another aspect of the linear regressions between PBE and dipole is the value of the critical dipole moment, from which we must obtain bound states of the positron with the cluster. The critical dipole moment can be determined from the smallest dipole in the ensemble of configurations and also through linear regression. According to the results of the linear regressions, the critical dipole moment should be 6.00D, 7.01D, and 7.41D for glycine clusters with 7, 14, and 30 water molecules. On the other hand, through the dipole moments of the ensemble, we found critical dipoles equal to 8.98D, 7.90D, and 5.65D for the same cluster sizes. Nummela^14^ reported critical dipole moments between 3.71 and 2.75D for glycine clusters with up to four water molecules, which diverged from our results due to the reduced number of water molecules. The divergence of the critical dipoles obtained via linear regression and from the ensemble results can be attributed to the low correlation between the PBE and the dipole. However, the increase in the cluster size results in a more significant variation of the dipole moment magnitudes for each cluster size. This large interval of dipole moments also contributes to the differences in the critical dipole moments obtained from linear regressions and the dipole moments of the ensemble of values. As PBE_KT_ and PBE_P2_ resulted in smaller correlation coefficients, we limited the discussion about critical dipole moments to the linear regressions of PBE_HF_.

### 3.3 Positron Orbitals

**Figure 3:**
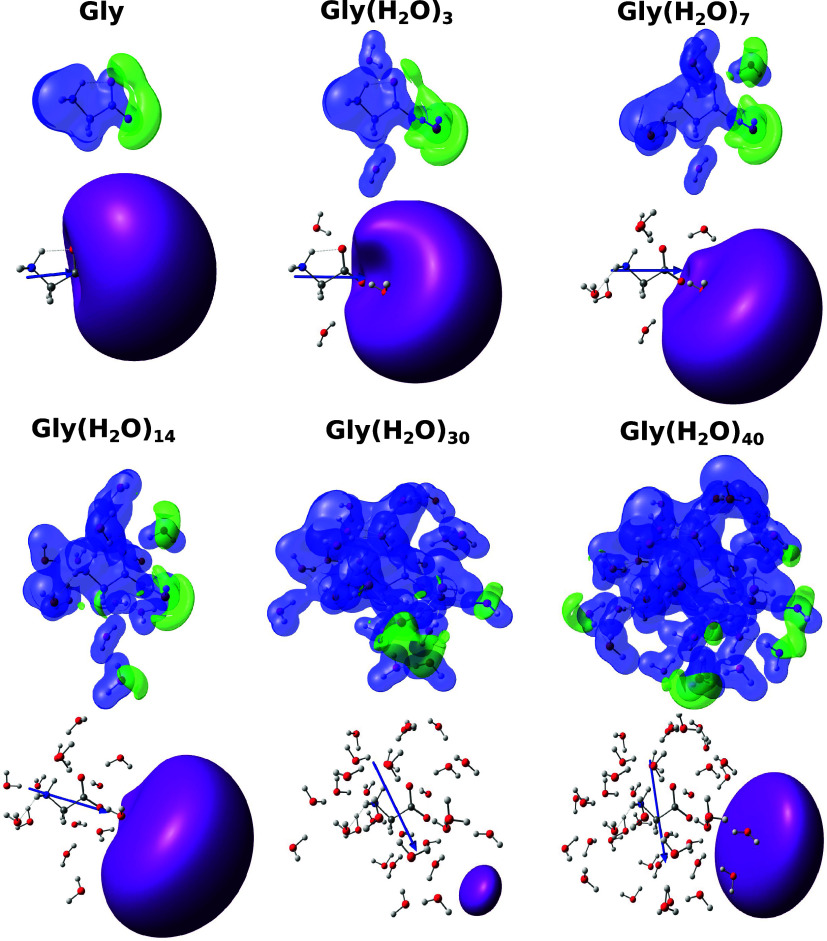
Electrostatic potential, dipole moment, and positron orbital for glycine isolated (Gly) and the clusters Gly(H_2_O)_3_, Gly(H_2_O)_7_, Gly(H_2_O)_14_, Gly(H_2_O)_30_ and Gly(H_2_O)_40_ obtained in APMO/HF calculations with the basis set combination 7s7p7*d*/6-31G++(d,p)/6-31G+(d,p). The electrostatic potential is represented in blue (positive) and green (negative) with isovalue 0.1. The positron orbital is represented in purple with isovalue 0.009.

The positron densities of the amino acid aggregates should provide important insights into positron interactions with solvated molecules and shed some light on the trends observed for the PBEs. Electrostatic potentials, dipole moments, and positron orbitals for glycine aggregates are shown in Figure 3. The clusters of different sizes were obtained from the same liquid configuration. Similar plots for alanine and proline are available as SI. For aggregates containing up to 3 solvent molecules, the regions with the lowest electrostatic potential are localized around the carboxylate groups, and hence around the negative pole of the solute. The shapes of the corresponding positronic orbitals resemble dipole-supported bound states. As the clusters grow larger, the low potential region tends to delocalize over the water molecules, especially on the water–vacuum boundary, which will be referred to as the surface. The delocalization should favor positron–solvent interactions, despite the negatively charged sites in the zwitterionic solutes. Although the shapes of the positronic orbitals still resemble dipole-supported states to some extent, they mostly localize on the cluster boundary. The low statistical correlation between the PBEs and dipole moments discussed above should be related to the competition between the formation of positron–solute and positron–solvent states. The first can be understood as solvated positronic molecules, while the latter as surface states.

The formation solute-water hydrogen bonds, in which the solvent molecule acts as donor, tends to stabilize electronic resonances (anion states formed by electron attachment).^68,69^ The same effect could be expected to disfavor positron attachment to hydrated amino acids. We calculated atomic charges for the oxygen atoms of the carboxylate groups using the CHELPG method^70^ along with HF densities. The charges were obtained without the positron and the same trends were observed for the three amino acids (see the SI), so we limit our discussion to the results for glycine. For the isolated species, the calculated charges were −0.7838 and −0.7788, where the oxygen atom that forms an intramolecular hydrogen bond with the methyl group is slightly more negative. For the glycine clusters with 40 water molecules, the charges are reduced to (−0.6875 ± 0.0162) and (−0.6502 ± 0.0167), where ensemble averages and standard error of the averages are indicated. The charges of the oxygen atoms of the water molecules follow the opposite trend, i.e., they increase in magnitude with the cluster size. The charge of oxygen atom of isolated water molecule is equal to −0.834 (force field charge), while for glycine clusters containing 40 water molecules, we found −0.9001 ± 0.0027. Therefore, the calculated CHELPG charges corroborate the more effective positron–solvent interactions in larger aggregates. The solute–solvent hydrogen bonds also attract water molecules to the vicinity of the amino acid, with positively charged hydrogen atoms (solvent) lying close to the negatively charged carboxylate groups (solute). The positron and positively charged atoms compete for the same site, namely, the carboxylate group vicinity, so the positron attachment to solvated amino acids is disfavored.

**Figure 4:**
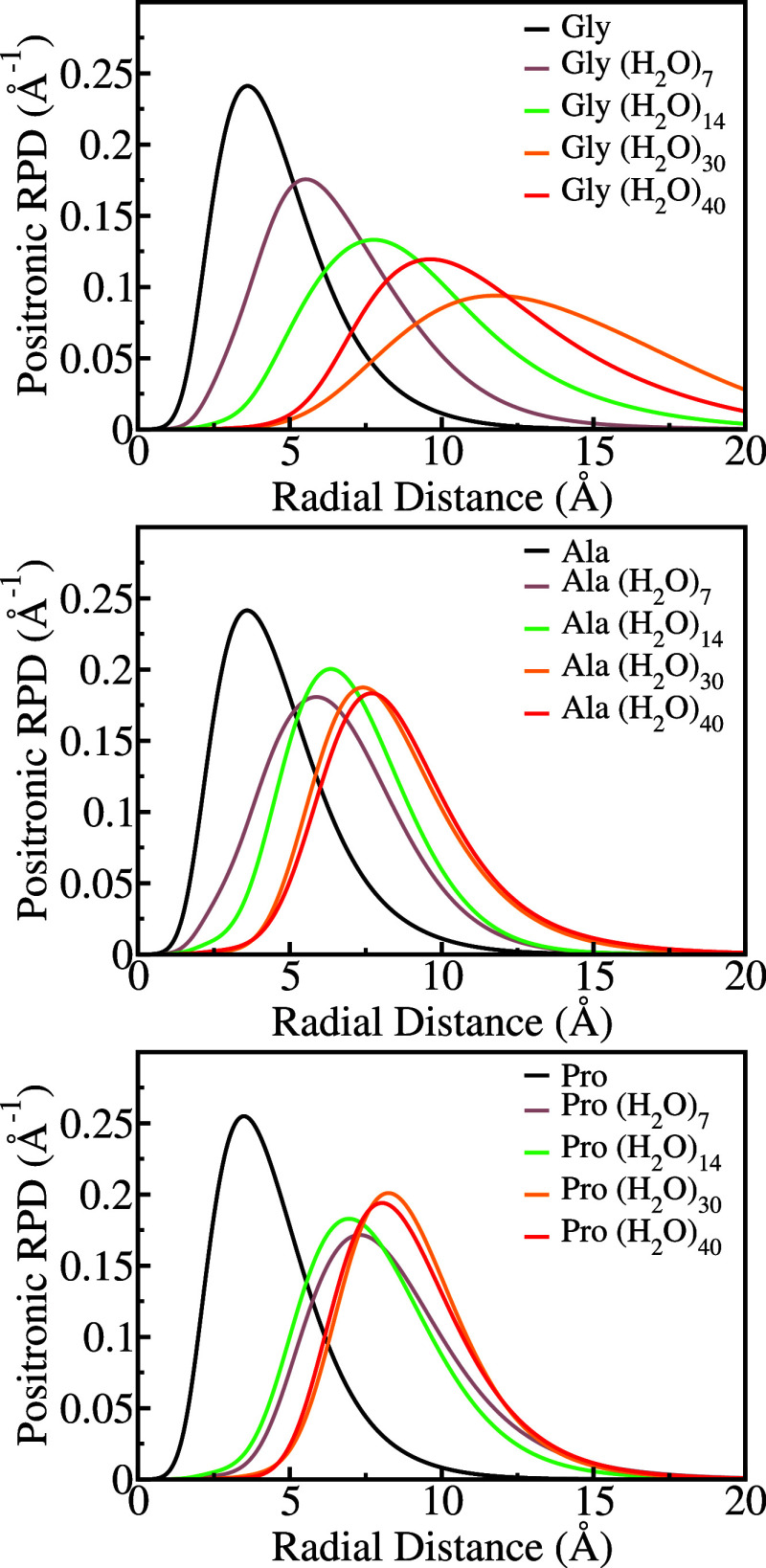
Positronic radial probability densities for glycine (upper panel), alanine (middle panel), and proline (bottom panel). The calculations were performed for clusters with 7 to 40 water molecules obtained from the same liquid configuration.

Further insight into positron localization in the amino acid–water aggregates can be gained from the radial distributions of the positronic orbitals. The radial probability densities (RPDs) of those orbitals, calculated as described in ref (40) for the same randomly chosen liquid configuration, are shown in Figure 4. For all systems, the origin of the RPD is located on the carbon atom of the carboxylate group (solute molecule). The probability densities are consistent with the discussion above, since their peaks tend to shift to larger distances as the number of solvent molecules increases, although not monotonically in all cases.

Table 3 presents the maximum values of the radial distribution (RDP_max_), the position of the maxima (*r*_max_) and full widths at half maximum (fwhm) averaged over the uncorrelated liquid configurations (results for other cluster sizes available as SI). Interestingly, the averaged peak positions increase monotonically with the cluster size for the three solutes. Comparing the largest clusters (30 water molecules) with the isolated amino acids, the mean peak positions are shifted by 4.94 Å, for glycine, 5.51 Å, for alanine, and 5.09 Å, for proline.

**Table 3:** Ensemble Averages for the Positronic RPD Maximum (RP*D*_max_), Full Width at Half Maximum (FWHM), and Maximum Position (*r*_max_) for Glycine (Gly), Alanine (Ala), and Proline (Pro)^a^

species	RPD_max_ (Å^–1^)	fwhm	*r*_max_ (Å)
Gly	0.24	3.75	3.60
Gly(H_2_O)_3_	0.19 ± 0.00	4.84 ± 0.09	4.95 ± 0.09
Gly(H_2_O)_7_	0.18 ± 0.00	5.11 ± 0.09	5.80 ± 0.09
Gly(H_2_O)_14_	0.18 ± 0.00	5.18 ± 0.11	6.58 ± 0.11
Gly(H_2_O)_30_	0.18 ± 0.00	5.58 ± 0.16	8.54 ± 0.13
Ala	0.24	3.77	3.60
Ala(H_2_O)_3_	0.19 ± 0.00	4.84 ± 0.10	4.89 ± 0.10
Ala(H_2_O)_7_	0.18 ± 0.00	5.31 ± 0.09	5.97 ± 0.11
Ala(H_2_O)_14_	0.17 ± 0.00	5.66 ± 0.12	6.84 ± 0.13
Ala(H_2_O)_30_	0.16 ± 0.00	5.97 ± 0.17	9.11 ± 0.17
Pro	0.25	3.57	3.48
Pro(H_2_O)_3_	0.20 ± 0.00	4.64 ± 0.08	4.84 ± 0.10
Pro(H_2_O)_7_	0.19 ± 0.00	4.95 ± 0.09	5.71 ± 0.12
Pro(H_2_O)_14_	0.18 ± 0.00	5.22 ± 0.13	6.40 ± 0.14
Pro(H_2_O)_30_	0.17 ± 0.00	5.78 ± 0.15	8.57 ± 0.17

a Calculations were performed for isolated and solvated amino acids with the 7s7p7*d*/6-31G++(d,p)/6-31G+(d,p) basis set combination. All results are presented with the respective standard error of the average, except for isolated amino acid results.

The observed shifts of the peaks, properly averaged over the ensemble of liquid configurations, further indicate that the positron density is driven toward the cluster surface. This process can be rationalized based on the results discussed so far. Hydrogen bonding between the solute and solvent molecules prevents positron localization around the negatively charged carboxylate groups. Positrons barely bind to individual water molecules, so they are pushed toward the cluster regions with negative electrostatic potential. As the clusters increase in size, they become more strongly polar (Table 1) with negative potentials around the surface (Figure 3). Another important aspect noted in the ensemble averages of peak positions is the larger shifts of alanine clusters in comparison with glycine and proline results. The less polar aggregates and more significant shifts observed for alanine clusters are evidence of more accentuated surface states, resulting in less correlation between PBEs and dipole moments for alanine clusters.

In extended purely electronic systems, reduced-size approximations may lead to unphysical delocalization of the charge density.^71^ This drawback can be remedied by including an electrostatic embedding (EE) around the cluster, i.e., by describing the far-lying solvent molecules with effective atomic charges. We performed QM/MM calculations, as described in ref (40), to investigate whether the EE would induce the localization of the positron density around the solute. Despite the size of the cluster (QM region), the positron densities remained delocalized around the cluster surface (i.e., the boundary between the QM and MM regions). We only present the result for Proline aggregate (see SI) which is representative of the other cases because the EE approximation would only be meaningful if the positron was localized away from the atomic charges (MM region). Nevertheless, the QM/MM results provide additional evidence that positron attachment to solvated molecules is suppressed, even for zwitterionic amino acids having carboxylate groups.

### 3.4 Annihilation Rates

**Figure 5:**
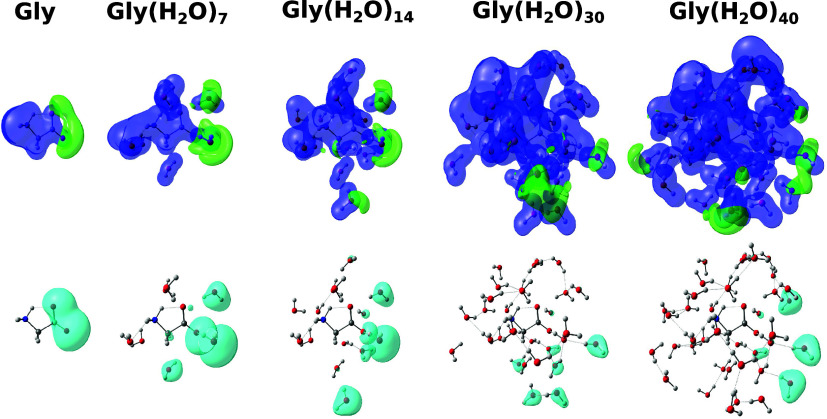
Electrostatic potential and contact density for isolated and hydrated glycine. The electrostatic potential is represented by blue (positive) and green (negative) isosurfaces with the isovalue 0.1 au, while contact density by cyan isosurface with the isovalue 8 × 10^–7^ au.

The annihilation rates obtained from APMO/HF calculations (Γ_HF_), using the 7s7p7*d*/6-31G++(d,p)/6-31G+(d,p) basis set combination, are shown in Table 4. Since the HF approximation is known to largely underestimate the rates, we also show results improved with enhancement factors (Γ_ef_), as described in Section 2.2. For isolated glycine, alanine, and proline, the Γ_HF_ estimates are, respectively, 10.02, 10.22, and 11.54 ns^–1^. The enhancement factors increase the annihilation rates by a factor of 3.5, such that Γ_ef_ = 34.69, 35.50, and 40.90 ns^–1^ for glycine, alanine, and proline. Ozaki et al.^29^ also reported HF-level rates between 1.22 and 1.73 ns^–1^ for amino acids in the hydrogen-bonded conformation. The use of enhancement factors provided improved rates ranging from 4.79 to 6.82 ns^–1^. The discrepancy between the theoretical estimates mainly arises from the different conformations, i.e., zwitterionic and hydrogen-bonded forms. The stronger positron attraction to the carboxylate group (zwitterions) favors the overlap between the electronic and positronic densities, thus leading to higher annihilation rates. The decomposition of the rates into valence and core (Γ_HF_^co^ and Γ_ef_^co^) contributions points out negligible contributions from the latter, around 1 and 0.1% for HF and improved annihilation rates, respectively. Further insight into annihilation is provided by the contact density isosurfaces shown in Figure 5 (see also the SI). As expected, the isolated amino acids present the contact densities around the carboxylate groups.

**Table 4:** Hartree–Fock Annihilation Rates (Γ_HF_), Contribution from Core Orbitals to the Hartree–Fock Rates (Γ_HF_^co^), Improved Estimates Obtained with Enhancement Factors (Γ_ef_), and the Core Contribution to the Improved Annihilation Rates (Γ_ef_^co^) for Isolated and Solvated Glycine (Gly), Alanine (Ala), and Proline (Pro)^a^

species	Γ_HF_ (10^–2^ ns^–1^)	Γ_HF_^co^ (10^–2^ ns^–1^)	Γ_ef_ (10^–2^ ns^–1^)	Γ_ef_^co^(10^–2^ ns^–1^)	*R*^2^
Gly	10.02	0.08	34.69	0.05	
Gly(H_2_O)_3_	8.94 ± 0.30	0.75 ± 0.04	34.33 ± 1.23	0.93 ± 0.05	0.71
Gly(H_2_O)_7_	9.01 ± 0.34	1.15 ± 0.04	32.92 ± 1.30	1.44 ± 0.05	0.60
Gly(H_2_O)_14_	9.83 ± 0.50	1.47 ± 0.07	35.64 ± 1.88	1.85 ± 0.09	0.65
Gly(H_2_O)_30_	10.41 ± 0.54	1.98 ± 0.12	36.77 ± 1.95	2.48 ± 0.16	0.76
Ala	10.22	0.08	35.50	0.05	
Ala(H_2_O)_3_	9.78 ± 0.34	0.82 ± 0.04	37.30 ± 1.37	1.03 ± 0.05	0.68
Ala(H_2_O)_7_	8.92 ± 0.39	1.11 ± 0.05	32.53 ± 1.47	1.39 ± 0.06	0.59
Ala(H_2_O)_14_	9.77 ± 0.67	1.44 ± 0.10	34.82 ± 2.40	1.81 ± 0.12	0.60
Ala(H_2_O)_30_	8.16 ± 0.63	1.45 ± 0.10	28.69 ± 2.26	1.81 ± 0.13	0.69
Pro	11.54	0.09	40.90	0.06	
Pro(H_2_O)_3_	9.66 ± 0.32	0.78 ± 0.04	37.46 ± 1.31	0.98 ± 0.05	0.79
Pro(H_2_O)_7_	9.29 ± 0.37	1.07 ± 0.04	34.69 ± 1.45	1.34 ± 0.05	0.76
Pro(H_2_O)_14_	10.06 ± 0.58	1.37 ± 0.07	36.93 ± 2.24	1.72 ± 0.09	0.71
Pro(H_2_O)_30_	9.16 ± 0.62	1.58 ± 0.10	32.59 ± 2.30	1.98 ± 0.12	0.68

a The correlation coefficient for the linear regressions of Γ_HF_ and PBE_HF_ is also shown. The results are indicated as ensemble averages and standard error of the average, except for isolated amino acids.

The larger clusters have smaller rates compared to the isolated amino acids. This change is, of course, related to the shift from positronic amino acid states to positronic surface states. It is also clear from Figure 5 that the positron density tends to delocalize around water molecules. Interestingly, the core contribution to Γ_HF_ is much more significant in the clusters, compared to the isolated amino acids. For the aggregates with 30 water molecules, core annihilation essentially arises from the overlap with 1s electrons of the solvent oxygen atoms, accounting for 17 to 19% of the HF-level rates (Table 4). Our previous study of hydrated Ps atoms also indicated much less significant core contributions to the HF-level pick-off annihilation rates,^40^ i.e., positron–water interactions would favor core processes compared to Ps-water interactions. Even through enhancement factors, the core contribution (Γ_ef_^co^) is still more expressive for large clusters, accounting for 6 to 7% of Γ_ef_ annihilation rates. The present results, even for the clusters with 40 water molecules, do not adequately describe the positron states in the liquid bulk since the surface states arise from the finite size of the systems. However, the estimates of Γ_HF_^co^ and Γ_ef_^co^ suggest a significant contribution of the 1s orbitals to the annihilation rates, which could be relevant to positron-based cancer treatments.^11^

**Figure 6:**
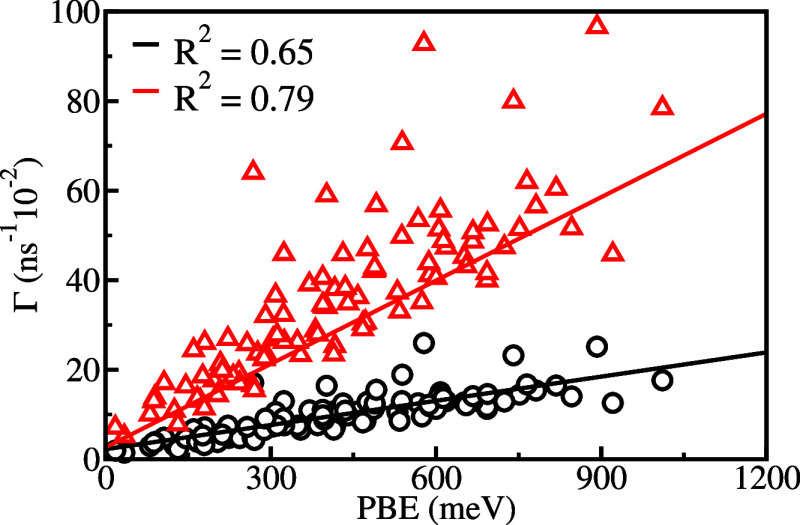
Linear regressions between Γ_HF_ (black circles) and Γ_ef_ (red triangles) with PBE_HF_ for Gly(H_2_O)_14_ clusters. The correlation coefficients (*R*^2^) are equal to 0.65 and 0.79 for Γ_HF_ and Γ_ef_, respectively. We considered clusters obtained from the set of statistically uncorrelated liquid configurations.

Recent studies of isolated molecules^29^ and binary clusters^32^ investigated the correlation between annihilation rates and the square root of the PBE. For the present systems, we found better correlation for Γ_HF_ as a linear function of PBE_HF_. The correlation coefficients are shown in Table 4 for Γ_HF_. In Figure 4, we also present the linear regressions of both Γ_HF_ and Γ_ef_ for glycine clusters composed of 14 water molecules, which are representative of the other cases. Although the coefficients are similar, better correlation was obtained for Γ_ef_ and PBE_HF_ in all cases. We further investigated the correlation between the annihilation rates with other cluster properties, such as dipole moment and polarizability, but the PBEs showed the stronger correlation (Figure 6).

## 4 Conclusions

We investigated solvent effects on the attachment of positrons to zwitterionic glycine, alanine, and proline using the sequential QM/MM method along with APMO calculations. For the isolated amino acids and small clusters, the positron density localizes around the negatively charged carboxylate group, which can be viewed as isolated or microsolvated positronic amino acid states. For larger clusters, composed of 6 to 40 water molecules, the formation of solvent–solute hydrogen bonds disfavors positron attachment to the solute molecules, so that positronic surface states are formed. We further included an electrostatic embedding around the larger clusters, but the classical field did not induce the localization of the positron around the solute. Positron interactions with biomolecules have attracted attention in the past decade or more.^72^ In particular, positron attachment to solvated positronic biomolecules could be expected to take place, in analogy to the formation of transient anions that can induce radiation damage.^41,42^ While our cluster models, even the larger ones, do not account for bulk properties, the present results suggest that hydrogen bonding would suppress positron attachment to solvated biomolecules, even zwitterionic amino acids having carboxylate groups. In addition, our calculations indicate a significant contribution of the core orbitals of water molecules to annihilation rates, which could be relevant to positron-based cancer treatments.^11^ Finally, we explored the correlations between PBEs and dipole moments, as well as annihilation rates and PBEs, consistent with previous studies for smaller clusters.^12−15^

## Supporting Information Available

Additional details about classical simulations, positron representation in quantum calculations, positron binding energies, positron orbitals, annihilation rates, electrostatic potentials, and contact densities (PDF)
