# Supplementary material for: Does Positron Attachment Take Place in Water Solution?
Source: J Phys Chem B. 2024 Oct 9;128(41):10178–88. doi: 10.1021/acs.jpcb.4c03627 (PMC11492259; doi:10.1021/acs.jpcb.4c03627)
Supplement: Supplementary file 1 — jp4c03627_si_001.pdf [file jp4c03627_si_001.pdf]

Supporting Information
"Does Positron Attachment Take Place in Water Solution?"

Mateus Bergami[1], Jorge Charry[2], Andres Reyes[3], Kaline Coutinho[1] and Márcio T. do N. Varella*[1]

[1]Instituto de Física, Universidade de São Paulo, Rua do Matão 1371, CEP 05508-090 São Paulo, SP, Brazil.
[2]Department of Physics and Materials Science, University of Luxembourg, L-1511 Luxembourg City, Luxembourg
[3]Department of Chemistry, Universidad Nacional de Colombia, Av. Cra. 30 #45-03, 111321, Bogotá, Colombia

1 Classical simulations

We performed classical Monte Carlo (MC) simulations in the NpT ensemble ($T = 298.15$ K and $p = 1$ atm) with 1000 water molecules and a single biomolecule in a cubic box. All molecules are rigid during the simulations. We considered glycine, alanine, and proline in zwitterionic form. The geometry and parameters of the SPC/E force field were employed for water molecules. At the same time, the biomolecule was described by the Lennard-Jones parameters of the OPLS/AA force field, and atomic charges were obtained using the CHELPG method and the polarizable continuum model.

Periodic boundary conditions and the image method were employed in the simulation box so that the molecules did not fill the edges of the box. A cutoff sphere, with a radius equal to 14.35 Å corresponding to 50% of the box size, is centered on each molecule. The sum of interactions due to all molecules outside the sphere is replaced by a long-range correction based on a continuum model.

The MC simulations were based on the Metropolis acceptance algorithm and performed with the DICE software. Thermalization was achieved afterwards 3×10^8 MC steps and the production phase consisted of 6×10^8 MC steps. We found an average density equal to $0.99 \pm 0.00\,\mathrm{g/cm^3}$ in agreement with the experimental result for liquid water.

*mvarella@if.usp.br

2 Hydrogen bonds

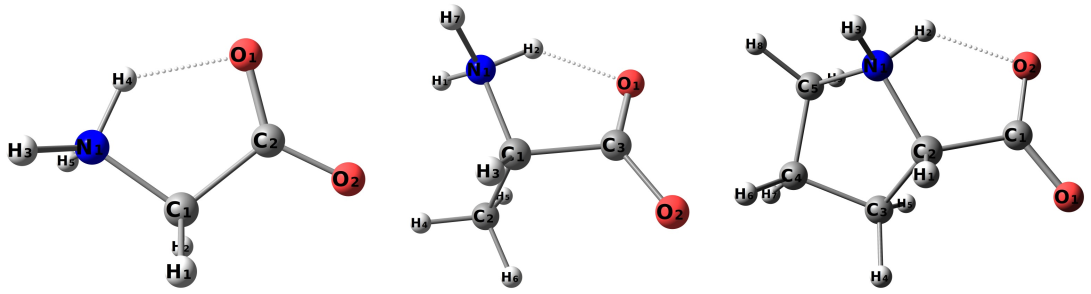

Figure S1: Optimized geometries of glycine, alanine, and proline molecules in the zwitterionic form. Oxygen atoms are represented in red, nitrogen in blue, carbon in gray, and hydrogen in white. Atoms are numbered by type.

The average number of hydrogen bonds on the atomic sites of each molecule was estimated by analyzing the statistics of acceptor and donor bonds of the solute and solvent molecules for all uncorrelated liquid configurations. We determined the formation of hydrogen bonds from geometric and energetic criteria. Labeling the donor and acceptor atoms D and A, respectively, we consider that an H-bond is formed in case the D−A distance is smaller than 3.55 Å for glycine and alanine, and 3.45 Å for proline. The geometric criteria also comprise the planar angle H−D−A, which can not exceed 40 degrees. In contrast, the energetic criteria impose energies smaller than -0.01 kcal/mol for H-bonds. Tab S1 presents the average number of hydrogen bonds and their associated energies.

Species	H-Bonds	$\langle N \rangle_{\mathrm{HB}}$	$\langle E \rangle_{\mathrm{HB}}$(kcal/mol)
Glycine	C=O···H−O	2.9	-8.98 ± 0.10
	C−O···H−O	2.4	-7.89 ± 0.12
	3×(N−H···O−H)	2	-9.75 ± 0.13
	C−N···O−H	0	
	sum	7.3	-26.62 ± 0.35
Alanine	C=O···H−O	2.7	-9.29 ± 0.10
	C−O···H−O	2.4	-8.38 ± 0.12
	3×(N−H···O−H)	2.3	-9.29 ± 0.09
	C−N···O−H	0	
	sum	7.4	-26.96 ± 0.31
Proline	C=O···H−O	3.1	-9.39 ± 0.08
	C−O···H−O	2.2	-8.29 ± 0.14
	2×(N−H···O−H)	1.3	-8.78 ± 0.17
	C−N···O−H	0	
	sum	6.6	-26.46 ± 0.39

Table S1: Statistical analyses of the average number of hydrogen bonds ($\langle N \rangle_{\mathrm{HB}}$) and their associated energies ($\langle E \rangle_{\mathrm{HB}}$) obtained from MC sampling.

3 Positronic expansion centers

The tests were carried out using different expansion centers for the positron, using 100 uncorrelated liquid configurations with the APMO/HF method. Different ways were used to represent the positron in quantum calculations; the first two ways consist of using a single positronic expansion center on one of the solute's oxygens (O1 and O2), the third consists of positioning the positronic center on the solute's nitrogen (N), the fourth uses centers on both oxygens (OO), and the last employ centers on the oxygens and nitrogen atoms (OON).

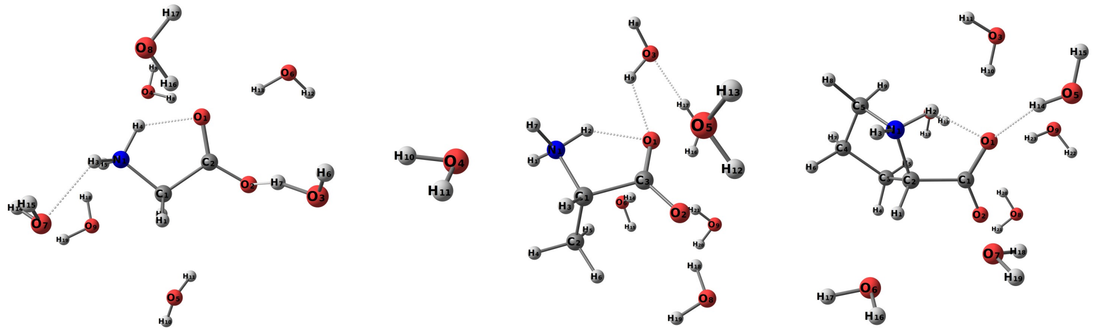

Figure S2: Liquid configurations composed of a solute molecule and water molecules of the first solvation shell. Oxygen atoms are represented in red, nitrogen in blue, carbon in gray, and hydrogen in white. Atoms are numbered by type. These liquid configurations belong to the set of uncorrelated liquid configurations.

Table S2 displays the ensemble averages of the positron binding energy (PBE_{HF}) and the annihilation rate (Γ_{HF}), based on the different positron representations. Positioning the positron expansion centers on oxygen atoms leads to a notable increase in both PBE_{HF} and Γ_{HF}. This increase occurs because the positron tends to associate with areas of the molecule with a negative charge density. Specifically, the oxygen atom not involved in the intramolecular hydrogen bond (O2) shows a solid propensity to bond with the positron. Therefore, the use of a single positronic expansion center yields PBE_{HF} and Γ_{HF} values that are in line with those obtained from centers on oxygens (OO) and all electronegative atoms (OON). Considering the significant computational resources required for multiple positronic expansion centers, we used a single expansion center on oxygen without intramolecular hydrogen bonding (O2) to test different basis sets.

	$Gly(H_2O)_7$		$Ala(H_2O)_7$		$Pro(H_2O)_7$	
Scheme	PBE_{HF}	Γ_{HF}	PBE_{HF}	Γ_{HF}	PBE_{HF}	Γ_{HF}
O1	307.4 ± 13.6	8.049 ± 0.319	252.3 ± 12.0	7.672 ± 0.332	334.6 ± 13.2	8.297 ± 0.319
O2	348.7 ± 15.1	9.116 ± 0.343	295.3 ± 13.6	8.503 ± 0.413	368.0 ± 14.6	9.082 ± 0.368
N1	220.8 ± 10.0	6.354 ± 0.209	167.1 ± 8.4	5.919 ± 0.230	226.5 ± 9.4	6.455 ± 0.210
OO	393.9 ± 15.9	9.116 ± 0.343	333.6 ± 14.2	8.921 ± 0.395	413.1 ± 15.9	9.127 ± 0.343
OON	413.3 ± 16.5	9.116 ± 0.343	353.4 ± 14.8	8.874 ± 0.372	429.4 ± 16.2	9.092 ± 0.339

Table S2: APMO/HF calculations with the 7s7p7d/6-31G++(d,p)/6-31G+(d,p) basis set combination for PBE_{HF} in meV and Γ_{HF} in $10^{-2}ns^{-1}$ obtained with different schemes to represent the positron through positronic expansion centers on solute's atoms. We employed positronic expansion centers on the oxygen with intramolecular hydrogen bond (O1), on the oxygen without intramolecular hydrogen bond (O2), on the nitrogen (N1), on both oxygens (OO), and on the three atoms simultaneously (OON).

The tests employed different sets of positronic basis, including 3s3p3d, 5s5p5d, 7s7p7d, 9s9p9d, 11s11p11d and 13s13p13d. For all calculations, we placed the electronic 6-31G++(d,p) basis set on solute atoms and 6-31G+(d,p) on solvent atoms. The data in Tables S3 and S4 suggest a convergence of PBE_{HF} and Γ_{HF} when using the 7s7p7d positronic basis set. Therefore, our study of positron attachment to glycine, alanine, and proline considered two positronic expansion centers on solute's oxygen atoms with the basis set 7s7p7d, electronic 6-31G++(d,p) basis set on solute atoms and 6-31G+(d,p) on solvent atoms.

	$Gly(H_2O)_7$		$Ala(H_2O)_7$	
Basis set	PBE_{HF}	Γ_{HF}	PBE_{HF}	Γ_{HF}
3s3p3d	-936.5 ± 43.5	57.951 ± 1.523	-916.1 ± 42.6	58.405 ± 1.533
5s5p5d	174.8 ± 1.6	11.465 ± 0.320	118.1 ± 14.0	11.772 ± 0.355
7s7p7d	307.4 ± 13.6	8.049 ± 0.319	252.3 ± 12.0	7.672 ± 0.332
9s9p9d	314.3 ± 13.3	7.629 ± 0.328	261.0 ± 11.6	7.155 ± 0.339
11s11p11d	314.4 ± 13.3	7.624 ± 0.328	261.1 ± 11.6	7.144 ± 0.340
13s13p13d	314.4 ± 13.3	7.623 ± 0.328	261.1 ± 11.6	7.144 ± 0.340

Table S3: APMO/HF calculations of PBE_{HF} in meV and Γ_{HF} in $10^{-2}ns^{-1}$ obtained with the positronic expansion center positioned on oxygen atom with intramolecular hydrogen bond (O1) of glycine and alanine. We employed the positronic basis sets 3s3p3d, 5s5p5d, 7s7p7d, 9s9p9d, 11s11p11d, and 13s13p13d. The electronic 6-31G++(d,p) basis set was placed on the solute atoms and 6-31G+(d,p) on solvent atoms.

	$Pro(H_2O)_7$	
Basis set	PBE_{HF}	Γ_{HF}
3s3p3d	-786.3 ± 48.2	48.569 ± 1.752
5s5p5d	226.2 ± 14.7	11.178 ± 0.319
7s7p7d	334.6 ± 13.2	8.297 ± 0.319
9s9p9d	339.5 ± 13.0	8.002 ± 0.326
11s11p11d	339.5 ± 13.0	7.999 ± 0.327
13s13p13d	339.5 ± 13.0	7.998 ± 0.327

Table S4: APMO/HF calculations of PBE_{HF} in meV and Γ_{HF} in $10^{-2}ns^{-1}$ obtained with the positronic expansion center positioned on oxygen atom with intramolecular hydrogen bond (O1) of proline. We employed the positronic basis sets 3s3p3d, 5s5p5d, 7s7p7d, 9s9p9d, 11s11p11d, and 13s13p13d. The electronic 6-31G++(d,p) basis set was positioned on solute atoms and 6-31G+(d,p) on solvent atoms.

4 Positron binding energy

Species	PBE_{HF}(meV)	PBE_{KT}(meV)	PBE_{P2}(meV)	$\langle\mu\rangle$ (Debye)	R_1^2	R_2^2	R_3^2
Gly	507.5	585.3	750.8	11.71			
$Gly(H_2O)_1$	450.1 ± 11.7	518.9 ± 13.3	657.5 ± 15.4	12.46 ± 0.14	0.30	0.22	0.12
$Gly(H_2O)_2$	421.9 ± 13.5	486.6 ± 15.5	607.6 ± 17.9	12.98 ± 0.20	0.38	0.29	0.18
$Gly(H_2O)_3$	402.5 ± 15.2	462.3 ± 17.4	567.3 ± 19.7	13.58 ± 0.24	0.36	0.27	0.17
$Gly(H_2O)_4$	384.7 ± 15.1	441.0 ± 17.4	536.2 ± 19.6	14.10 ± 0.24	0.42	0.33	0.22
$Gly(H_2O)_5$	377.1 ± 15.2	429.6 ± 17.3	513.5 ± 19.0	14.82 ± 0.25	0.52	0.43	0.33
$Gly(H_2O)_6$	382.1 ± 14.3	434.2 ± 16.4	511.6 ± 17.6	15.45 ± 0.26	0.53	0.44	0.35
$Gly(H_2O)_7$	393.9 ± 15.9	447.5 ± 18.1	522.5 ± 19.4	15.91 ± 0.31	0.60	0.51	0.44
$Gly(H_2O)_{14}$	420.0 ± 22.1	476.8 ± 25.0	533.2 ± 26.5	18.07 ± 0.46	0.63	0.57	0.53
$Gly(H_2O)_{30}$	421.4 ± 27.2	467.5 ± 29.7		23.68 ± 0.92	0.73	0.71	

Table S5: Positron binding energies obtained from APMO/HF (PBE_{HF}), APMO/Koopmans' theorem (PBE_{KT}), and APMO/P2 (PBE_{P2}) calculations for glycine (Gly) in the zwitterionic form. The HF-level dipole moments ($\langle\mu\rangle$) and correlation coefficients R_1^2, R_2^2, and R_3^2 for linear regressions between PBE_{HF}, PBE_{KT}, and PBE_{P2} with $\langle\mu\rangle$ are also shown. Calculations were performed for isolated and solvated amino acid with the 7s7p7d/6-31G++(d,p)/6-31G+(d,p) basis set combination. All values correspond to the averages over all 100 configurations. The results are presented with the respective standard error of the average, except for the isolated amino acid results.

Species	PBE_{HF}(meV)	PBE_{KT}(meV)	PBE_{P2}(meV)	$\langle\mu\rangle$ (Debye)	R_1^2	R_2^2	R_3^2
Ala	498.9	585.6	754.7	11.35			
$Ala(H_2O)_1$	441.9 ± 11.6	518.9 ± 13.8	664.2 ± 16.7	11.77 ± 0.14	0.05	0.02	0
$Ala(H_2O)_2$	416.6 ± 14.9	486.8 ± 17.7	613.9 ± 21.2	12.46 ± 0.18	0.18	0.12	0.06
$Ala(H_2O)_3$	392.8 ± 14.5	458.4 ± 17.4	573.9 ± 20.8	12.85 ± 0.21	0.18	0.11	0.06
$Ala(H_2O)_4$	371.4 ± 13.6	433.7 ± 16.0	539.2 ± 18.6	13.41 ± 0.26	0.25	0.15	0.07
$Ala(H_2O)_5$	352.7 ± 14.5	410.3 ± 16.9	505.2 ± 19.3	13.82 ± 0.28	0.22	0.14	0.06
$Ala(H_2O)_6$	345.0 ± 14.6	398.6 ± 17.0	482.7 ± 19.1	14.42 ± 0.29	0.25	0.16	0.09
$Ala(H_2O)_7$	333.6 ± 14.2	385.4 ± 16.6	463.6 ± 18.6	14.81 ± 0.32	0.32	0.21	0.13
$Ala(H_2O)_{14}$	311.3 ± 18.7	363.6 ± 22.1	423.0 ± 24.2	15.19 ± 0.47	0.51	0.46	0.43
$Ala(H_2O)_{30}$	276.8 ± 20.9	312.2 ± 23.7		18.49 ± 0.72	0.42	0.36	

Table S6: Positron binding energies obtained from APMO/HF (PBE_{HF}), APMO/Koopmans' theorem (PBE_{KT}), and APMO/P2 (PBE_{P2}) calculations for alanine (Ala) in the zwitterionic form. The HF-level dipole moments ($\langle\mu\rangle$) and correlation coefficients R_1^2, R_2^2, and R_3^2 for linear regressions between PBE_{HF}, PBE_{KT}, and PBE_{P2} with $\langle\mu\rangle$ are also shown. Calculations were performed for isolated and solvated amino acid with the 7s7p7d/6-31G++(d,p)/6-31G+(d,p) basis set combination. All values correspond to the averages over all 100 configurations. The results are presented with the respective standard error of the average, except for the isolated amino acid results.

Species	PBE_{HF}(meV)	PBE_{KT}(meV)	PBE_{P2}(meV)	$\langle\mu\rangle$ (Debye)	R_1^2	R_2^2	R_3^2
Pro	577.8	684.4	866.1	12.11			
$Pro(H_2O)_1$	501.4 ± 10.7	594.4 ± 12.5	743.4 ± 14.3	12.82 ± 0.14	0.06	0.02	0
$Pro(H_2O)_2$	455.2 ± 13.5	537.9 ± 15.9	664.1 ± 18.1	13.38 ± 0.20	0.20	0.13	0.06
$Pro(H_2O)_3$	443.5 ± 15.3	519.0 ± 18.3	626.2 ± 20.5	14.34 ± 0.23	0.22	0.15	0.09
$Pro(H_2O)_4$	429.7 ± 16.1	499.8 ± 19.2	594.4 ± 21.4	14.84 ± 0.25	0.19	0.12	0.07
$Pro(H_2O)_5$	411.7 ± 15.3	478.4 ± 18.3	563.4 ± 20.4	15.18 ± 0.28	0.20	0.12	0.08
$Pro(H_2O)_6$	413.1 ± 15.9	478.7 ± 18.8	560.1 ± 20.7	15.65 ± 0.31	0.24	0.15	0.10
$Pro(H_2O)_7$	417.4 ± 17.2	482.6 ± 20.3	560.1 ± 22.4	16.13 ± 0.34	0.30	0.20	0.15
$Pro(H_2O)_{14}$	428.3 ± 25.9	493.7 ± 30.0	556.2 ± 31.6	17.76 ± 0.52	0.50	0.44	0.40
$Pro(H_2O)_{30}$	390.4 ± 28.5	434.7 ± 31.4		22.60 ± 0.96	0.71	0.66	

Table S7: Positron binding energies obtained from APMO/HF (PBE_{HF}), APMO/Koopmans' theorem (PBE_{KT}), and APMO/P2 (PBE_{P2}) calculations for proline (Pro) in the zwitterionic form. The HF-level dipole moments ($\langle\mu\rangle$) and correlation coefficients R_1^2, R_2^2, and R_3^2 for linear regressions between PBE_{HF}, PBE_{KT}, and PBE_{P2} with $\langle\mu\rangle$ are also shown. Calculations were performed for isolated and solvated amino acid with the 7s7p7d/6-31G++(d,p)/6-31G+(d,p) basis set combination. All values correspond to the averages over all 100 configurations. The results are presented with the respective standard error of the average, except for the isolated amino acid results.

Species	PBE_{KT}(meV)	$\mathcal{T}_{ORX}$(meV)	$\mathcal{T}_{PRM}$(meV)
Gly	585.3	-65.1	230.7
$Gly(H_2O)_1$	518.9 ± 13.3	-58.3 ± 1.7	196.8 ± 4.5
$Gly(H_2O)_2$	486.6 ± 15.5	-55.0 ± 2.1	176.0 ± 5.6
$Gly(H_2O)_3$	462.3 ± 17.4	-51.1 ± 2.4	156.2 ± 6.1
$Gly(H_2O)_4$	441.0 ± 17.4	-48.4 ± 2.7	143.6 ± 6.1
$Gly(H_2O)_5$	429.6 ± 17.3	-45.3 ± 2.6	129.2 ± 5.4
$Gly(H_2O)_6$	434.2 ± 16.4	-45.1 ± 2.5	122.4 ± 4.8
$Gly(H_2O)_7$	447.5 ± 18.1	-46.5 ± 2.7	121.5 ± 5.1
$Gly(H_2O)_{14}$	476.8 ± 25.0	-49.6 ± 3.5	106.0 ± 5.9

Table S8: Decomposition of the APMO/P2 positron binding energy into the Koopmans (PBE_{KT}), relaxation ($\mathcal{T}_{ORX}$) and correlation ($\mathcal{T}_{PRM}$) contributions for glycine (Gly) in the zwitterionic form. Calculations were performed for isolated and solvated amino acid with the 7s7p7d/6-31G++(d,p)/6-31G+(d,p) basis set combination. All results are presented with the corresponding standard error of the average, except for isolated amino acid results.

Species	PBE_{KT}(meV)	$\mathcal{T}_{ORX}$(meV)	$\mathcal{T}_{PRM}$(meV)
Ala	585.6	−73.8	242.9
Ala$(H_2O)_1$	518.9 ± 13.8	−66.0 ± 2.4	211.3 ± 5.8
Ala$(H_2O)_2$	486.8 ± 17.7	−60.8 ± 2.9	187.8 ± 7.0
Ala$(H_2O)_3$	458.4 ± 17.4	−57.1 ± 3.1	172.5 ± 7.2
Ala$(H_2O)_4$	433.7 ± 16.0	−54.4 ± 3.0	160.0 ± 6.7
Ala$(H_2O)_5$	410.3 ± 16.9	−50.5 ± 3.0	145.4 ± 6.4
Ala$(H_2O)_6$	398.6 ± 17.0	−47.0 ± 3.0	131.1 ± 6.3
Ala$(H_2O)_7$	385.4 ± 16.6	−45.3 ± 3.0	123.6 ± 6.2
Ala$(H_2O)_{14}$	363.6 ± 22.1	−46.3 ± 3.9	105.7 ± 6.7

Table S9: Decomposition of the APMO/P2 positron binding energy into the Koopmans (PBE_{KT}), relaxation ($\mathcal{T}_{ORX}$) and correlation ($\mathcal{T}_{PRM}$) contributions for Alanine (Ala) in the zwitterionic form. Calculations were performed for isolated and solvated amino acid with the 7s7p7d/6-31G++(d,p)/6-31G+(d,p) basis set combination. All results are presented with the corresponding standard error of the average, except for isolated amino acid results.

Species	PBE_{KT}(meV)	$\mathcal{T}_{ORX}$(meV)	$\mathcal{T}_{PRM}$(meV)
Pro	684.4	−88.4	270.2
Pro$(H_2O)_1$	594.4 ± 12.5	−78.3 ± 2.0	227.3 ± 4.6
Pro$(H_2O)_2$	537.9 ± 15.9	−70.1 ± 2.6	196.2 ± 5.8
Pro$(H_2O)_3$	519.0 ± 18.3	−64.2 ± 3.2	171.5 ± 6.3
Pro$(H_2O)_4$	499.8 ± 19.2	−60.0 ± 3.4	154.6 ± 6.5
Pro$(H_2O)_5$	478.4 ± 18.3	−57.2 ± 3.4	142.2 ± 6.4
Pro$(H_2O)_6$	478.7 ± 18.8	−56.5 ± 3.5	137.9 ± 6.4
Pro$(H_2O)_7$	482.6 ± 20.3	−56.1 ± 3.7	133.7 ± 6.7
Pro$(H_2O)_{14}$	493.7 ± 30.0	−57.3 ± 4.9	119.8 ± 7.3

Table S10: Decomposition of the APMO/P2 positron binding energy into the Koopmans (PBE_{KT}), relaxation ($\mathcal{T}_{ORX}$) and correlation ($\mathcal{T}_{PRM}$) contributions for Proline (Pro) in the zwitterionic form. Calculations were performed for isolated and solvated amino acid with the 7s7p7d/6-31G++(d,p)/6-31G+(d,p) basis set combination. All results are presented with the corresponding standard error of the average, except for isolated amino acid results.

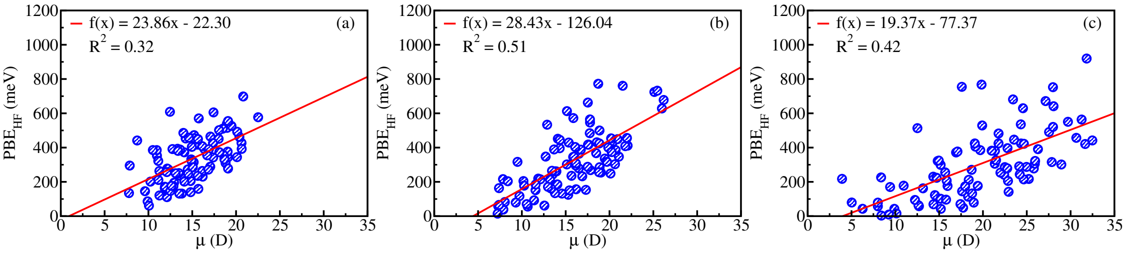

Figure S3: Linear regressions between the HF-level positron binding energies (PBE_{HF}) and dipole moments (μ) for alanine clusters: (a) Ala(H_2O)$_7$; (b) Ala(H_2O)$_{14}$; (c) Ala(H_2O)$_{30}$. The linear regressions were performed for the sets of uncorrelated liquid configurations.

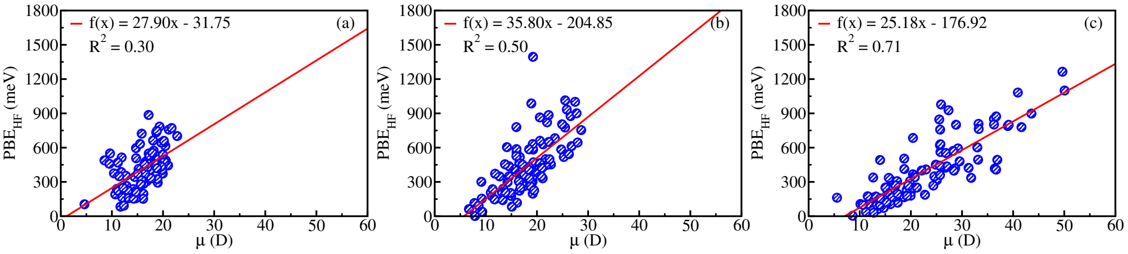

Figure S4: Linear regressions between the HF-level positron binding energies (PBE_{HF}) and dipole moments (μ) for proline clusters: (a) Pro(H_2O)$_7$; (b) Pro(H_2O)$_{14}$; (c) Pro(H_2O)$_{30}$. The linear regressions were performed for the sets of uncorrelated liquid configurations.

5 Positron orbitals

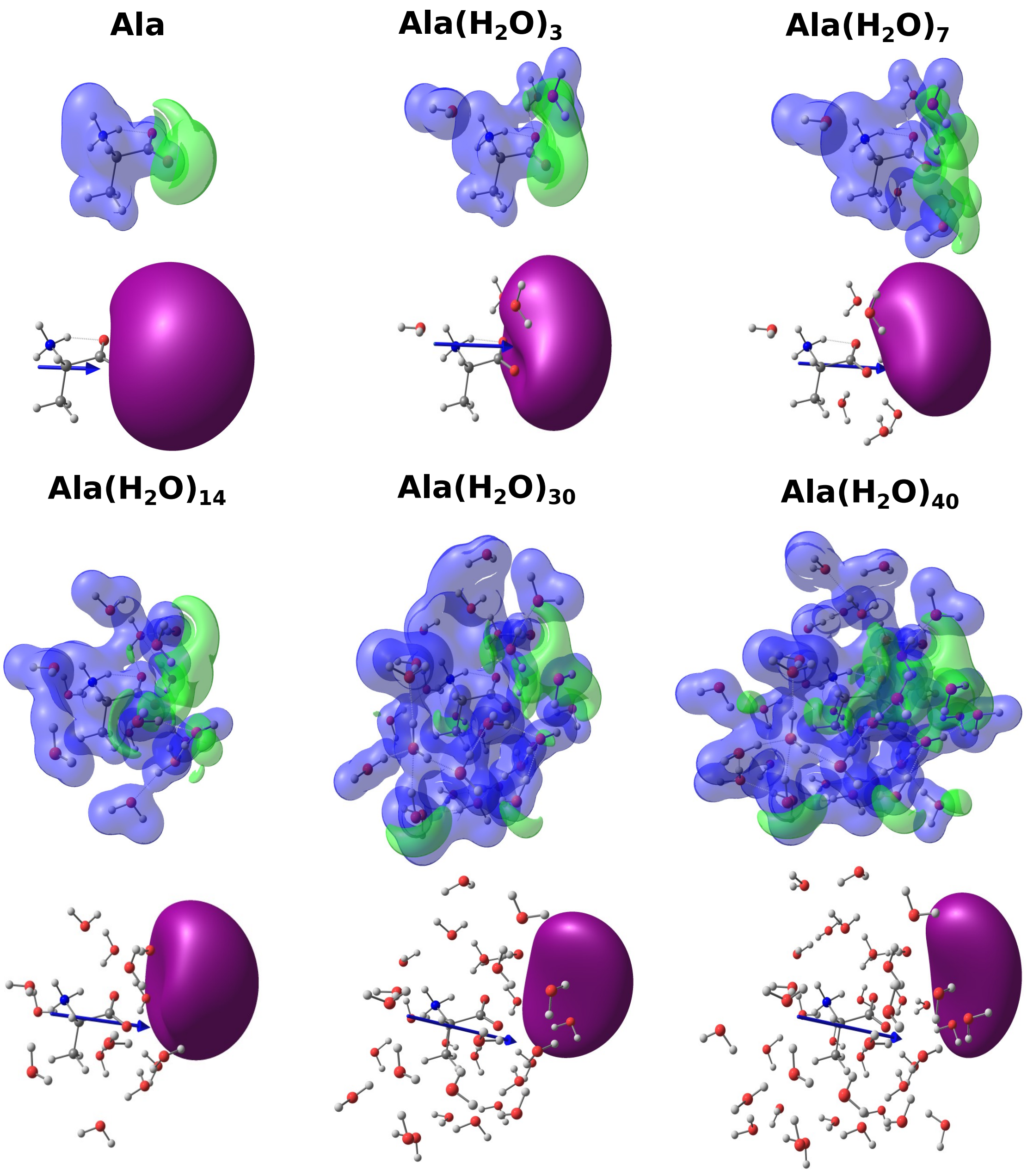

Figure S5: Electrostatic potential, dipole moment, and positron orbital for alanine isolated (Ala) and the clusters $Ala(H_2O)_3$, $Ala(H_2O)_7$, $Ala(H_2O)_{14}$, $Ala(H_2O)_{30}$ and $Ala(H_2O)_{40}$ obtained in APMO/HF calculations with the basis set combination 7s7p7d/6-31G++(d,p)/6-31G+(d,p). The electrostatic potential is represented in blue (positive) and green (negative) with isovalue 0.1. The positron orbital is represented in purple with isovalue 0.015.

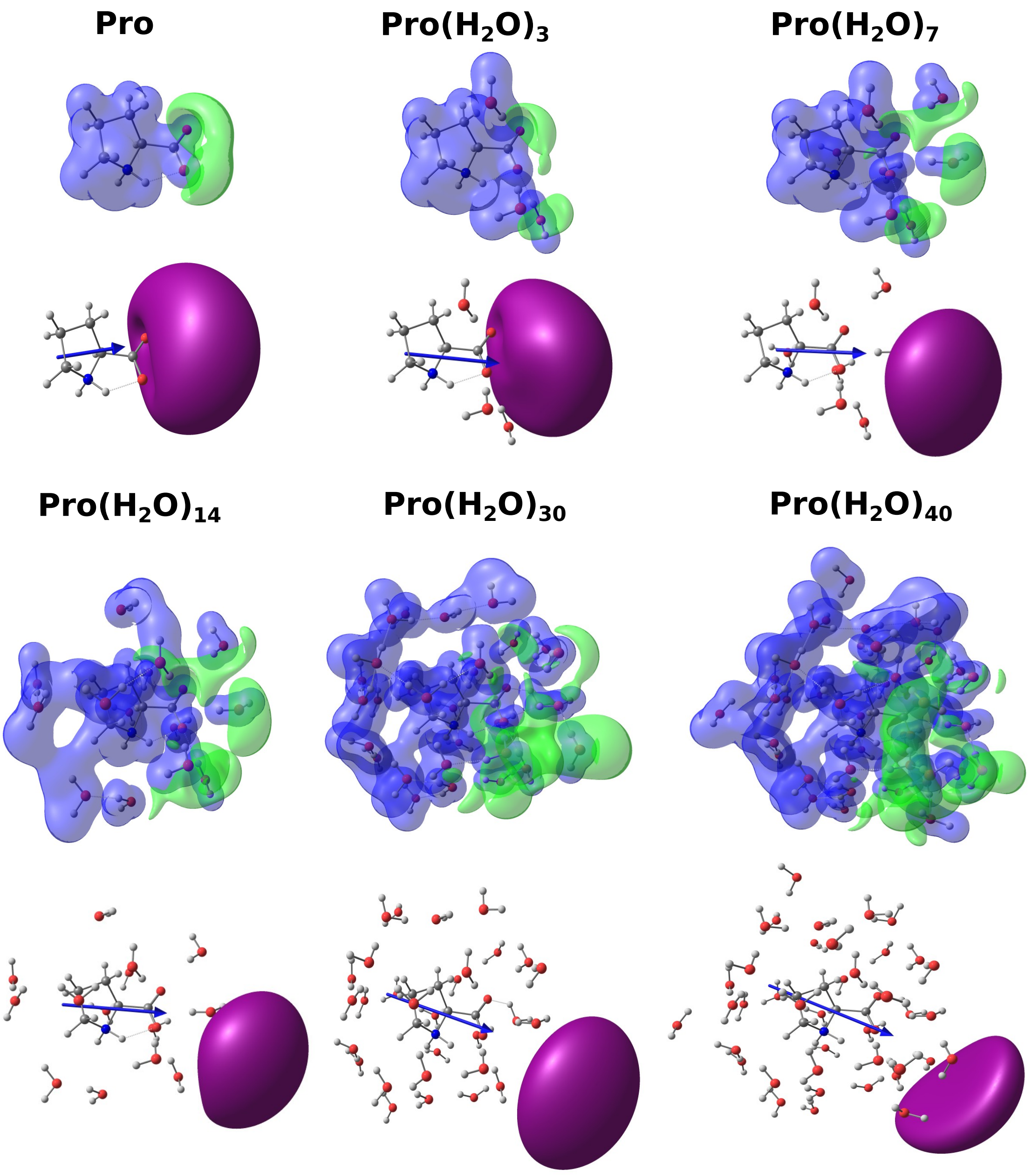

Figure S6: Electrostatic potential, dipole moment, and positron orbital for proline isolated (Pro) and the clusters Pro(H_2O)$_3$, Pro(H_2O)$_7$, Pro(H_2O)$_{14}$, Pro(H_2O)$_{30}$ and Pro(H_2O)$_{40}$ obtained in APMO/HF calculations with the basis set combination 7s7p7d/6-31G++(d,p)/6-31G+(d,p). The electrostatic potential is represented in blue (positive) and green (negative) with isovalue 0.1. The positron orbital is represented in purple with isovalue 0.015.

Species	O1	O2	O solv.
Gly	-0.7838	-0.7788	
$Gly(H_2O)_3$	-0.7350 ± 0.0069	-0.7122 ± 0.0092	-0.8520 ± 0.0041
$Gly(H_2O)_7$	-0.6767 ± 0.0102	-0.6543 ± 0.0141	-0.8436 ± 0.0028
$Gly(H_2O)_{14}$	-0.6429 ± 0.0164	-0.6068 ± 0.0183	-0.8652 ± 0.0026
$Gly(H_2O)_{30}$	-0.6626 ± 0.0165	-0.6231 ± 0.0178	-0.8955 ± 0.0023
$Gly(H_2O)_{40}$	-0.6875 ± 0.0162	-0.6502 ± 0.0167	-0.9001 ± 0.0027
Ala	-0.7698	-0.7523	
$Ala(H_2O)_3$	-0.7430 ± 0.0066	-0.7355 ± 0.0065	-0.8545 ± 0.0036
$Ala(H_2O)_7$	-0.6739 ± 0.0110	-0.6856 ± 0.0109	-0.8469 ± 0.0026
$Ala(H_2O)_{14}$	-0.6349 ± 0.0145	-0.6558 ± 0.0138	-0.8647 ± 0.0024
$Ala(H_2O)_{30}$	-0.6481 ± 0.0148	-0.6808 ± 0.0130	-0.8960 ± 0.0022
$Ala(H_2O)_{40}$	-0.6677 ± 0.0159	-0.6894 ± 0.0128	-0.9020 ± 0.0026
Pro	-0.7680	-0.7649	
$Pro(H_2O)_3$	-0.7236 ± 0.0081	-0.7218 ± 0.0077	-0.8584 ± 0.0047
$Pro(H_2O)_7$	-0.6655 ± 0.0123	-0.6698 ± 0.0143	-0.8449 ± 0.0030
$Pro(H_2O)_{14}$	-0.6582 ± 0.0139	-0.6383 ± 0.0157	-0.8669 ± 0.0025
$Pro(H_2O)_{30}$	-0.6570 ± 0.0136	-0.6519 ± 0.0154	-0.9058 ± 0.0024
$Pro(H_2O)_{40}$	-0.6704 ± 0.0144	-0.6802 ± 0.0153	-0.8969 ± 0.0046

Table S11: Ensemble averages of atomic charge of solute's oxygen with intramolecular hydrogen bond (O1), without intramolecular hydrogen bond (O2), and oxygen atoms from water molecules (O solv.). We considered isolated amino acids and clusters with different numbers of water molecules. The calculations employed the CHELPG method at the Hartree-Fock level without the positron inclusion.

Species	RPD_{max} (Å^{-1})	FWHM	r_{max} (Å)
Gly	0.24	3.75	3.60
$Gly(H_2O)_1$	0.22 ± 0.00	4.29 ± 0.06	4.13 ± 0.06
$Gly(H_2O)_2$	0.20 ± 0.00	4.58 ± 0.07	4.51 ± 0.08
$Gly(H_2O)_3$	0.19 ± 0.00	4.84 ± 0.09	4.95 ± 0.09
$Gly(H_2O)_4$	0.19 ± 0.00	5.02 ± 0.09	5.23 ± 0.10
$Gly(H_2O)_5$	0.18 ± 0.00	5.14 ± 0.09	5.54 ± 0.09
$Gly(H_2O)_6$	0.18 ± 0.00	5.15 ± 0.09	5.70 ± 0.08
$Gly(H_2O)_7$	0.18 ± 0.00	5.11 ± 0.09	5.80 ± 0.09
$Gly(H_2O)_{14}$	0.18 ± 0.00	5.18 ± 0.11	6.58 ± 0.11
$Gly(H_2O)_{30}$	0.18 ± 0.00	5.58 ± 0.16	8.54 ± 0.13

Table S12: Ensemble averages for positronic RPD maximum (RPD_{max}), full width at half maximum (FWHM), and maximum position (r_{max}) for glycine isolated (Gly) and the clusters $Gly(H_2O)_1$, $Gly(H_2O)_2$, $Gly(H_2O)_3$, $Gly(H_2O)_4$, $Gly(H_2O)_5$, $Gly(H_2O)_6$, $Gly(H_2O)_7$, $Gly(H_2O)_{14}$, and $Gly(H_2O)_{30}$. All results are presented with the respective standard error of the average, except for the isolated amino acid results.

Species	$RPD_{max}(\text{Å}^{-1})$	FWHM	$r_{max}(\text{Å})$
Ala	0.24	3.77	3.60
$Ala(H_2O)_1$	0.22 ± 0.00	4.24 ± 0.06	4.13 ± 0.08
$Ala(H_2O)_2$	0.20 ± 0.00	4.61 ± 0.08	4.57 ± 0.09
$Ala(H_2O)_3$	0.19 ± 0.00	4.84 ± 0.10	4.89 ± 0.10
$Ala(H_2O)_4$	0.19 ± 0.00	4.98 ± 0.09	5.15 ± 0.10
$Ala(H_2O)_5$	0.18 ± 0.00	5.19 ± 0.10	5.46 ± 0.10
$Ala(H_2O)_6$	0.18 ± 0.00	5.24 ± 0.09	5.76 ± 0.11
$Ala(H_2O)_7$	0.18 ± 0.00	5.31 ± 0.09	5.97 ± 0.11
$Ala(H_2O)_{14}$	0.17 ± 0.00	5.66 ± 0.12	6.84 ± 0.13
$Ala(H_2O)_{30}$	0.16 ± 0.00	5.97 ± 0.17	9.11 ± 0.17

Table S13: Ensemble averages for positronic RPD maximum (RPD_{max}), full width at half maximum (FWHM), and maximum position (r_{max}) for alanine isolated (Ala) and the clusters $Ala(H_2O)_1$, $Ala(H_2O)_2$, $Ala(H_2O)_3$, $Ala(H_2O)_4$, $Ala(H_2O)_5$, $Ala(H_2O)_6$, $Ala(H_2O)_7$, $Ala(H_2O)_{14}$, and $Ala(H_2O)_{30}$. All results are presented with the respective standard error of the average, except for isolated amino acid results.

Species	$RPD_{max}(\text{Å}^{-1})$	FWHM	$r_{max}(\text{Å})$
Pro	0.25	3.57	3.48
$Pro(H_2O)_1$	0.23 ± 0.00	4.08 ± 0.06	3.99 ± 0.06
$Pro(H_2O)_2$	0.21 ± 0.00	4.42 ± 0.06	4.41 ± 0.08
$Pro(H_2O)_3$	0.20 ± 0.00	4.64 ± 0.08	4.84 ± 0.10
$Pro(H_2O)_4$	0.20 ± 0.00	4.80 ± 0.09	5.18 ± 0.10
$Pro(H_2O)_5$	0.19 ± 0.00	4.92 ± 0.10	5.44 ± 0.11
$Pro(H_2O)_6$	0.19 ± 0.00	4.94 ± 0.09	5.58 ± 0.12
$Pro(H_2O)_7$	0.19 ± 0.00	4.95 ± 0.09	5.71 ± 0.12
$Pro(H_2O)_{14}$	0.18 ± 0.00	5.22 ± 0.13	6.40 ± 0.14
$Pro(H_2O)_{30}$	0.17 ± 0.00	5.78 ± 0.15	8.57 ± 0.17

Table S14: Ensemble averages for positronic RPD maximum (RPD_{max}), full width at half maximum (FWHM), and maximum position (r_{max}) for proline isolated (Pro) and the clusters $Pro(H_2O)_1$, $Pro(H_2O)_2$, $Pro(H_2O)_3$, $Pro(H_2O)_4$, $Pro(H_2O)_5$, $Pro(H_2O)M_6$, $Pro(H_2O)_7$, $Pro(H_2O)_{14}$, and $Pro(H_2O)_{30}$. All results are presented with the respective standard error of the average, except for isolated amino acid results.

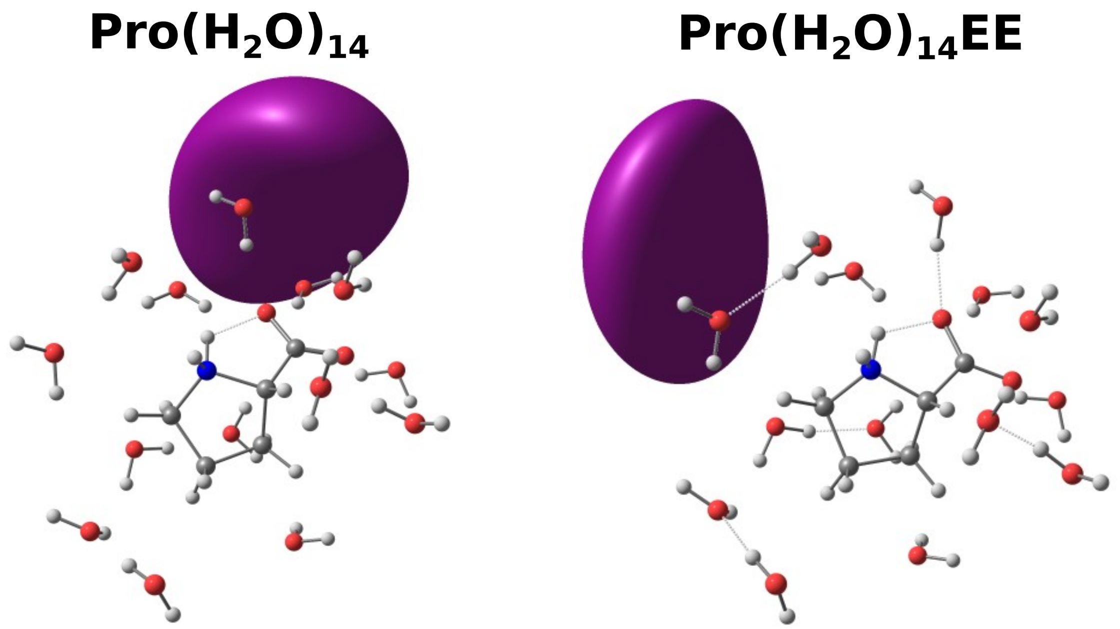

Figure S7: Positron orbitals of proline aggregate isolated Pro$(H_2O)_{14}$ and embedded Pro$(H_2O)_{14}$EE obtained in APMO/HF calculations with the basis set combination 7s7p7d/6-31G++(d,p)/6-31G+(d,p). The positron orbital is represented in purple with isovalue 0.015.

6 Annihilation rates

Species	$\Gamma_{HF}(10^{-2}ns^{-1})$	$\Gamma_{HF}^{co}(10^{-2}ns^{-1})$	$\Gamma_{ef}(10^{-2}ns^{-1})$	$\Gamma_{ef}^{co}(10^{-2}ns^{-1})$	R^2
Gly	10.02	0.08	34.69	0.05	
$Gly(H_2O)_1$	9.37 ± 0.21	0.38 ± 0.03	37.41 ± 0.87	0.47 ± 0.04	0.73
$Gly(H_2O)_2$	9.03 ± 0.26	0.56 ± 0.03	35.43 ± 1.10	0.70 ± 0.04	0.65
$Gly(H_2O)_3$	8.94 ± 0.30	0.75 ± 0.04	34.33 ± 1.23	0.93 ± 0.05	0.71
$Gly(H_2O)_4$	8.76 ± 0.32	0.86 ± 0.04	33.08 ± 1.27	1.08 ± 0.05	0.65
$Gly(H_2O)_5$	8.58 ± 0.31	0.97 ± 0.04	31.90 ± 1.23	1.22 ± 0.05	0.65
$Gly(H_2O)_6$	8.59 ± 0.28	1.06 ± 0.04	31.54 ± 1.11	1.33 ± 0.04	0.62
$Gly(H_2O)_7$	9.01 ± 0.34	1.15 ± 0.04	32.92 ± 1.30	1.44 ± 0.05	0.60
$Gly(H_2O)_{14}$	9.83 ± 0.50	1.47 ± 0.07	35.64 ± 1.88	1.85 ± 0.09	0.65
$Gly(H_2O)_{30}$	10.41 ± 0.54	1.98 ± 0.12	36.77 ± 1.95	2.48 ± 0.16	0.76

Table S15: Hartree-Fock annihilation rates (Γ_{HF}), contribution from core orbitals to the Hartree-Fock rates (Γ_{HF}^{co}), improved estimates obtained with enhancement factors (Γ_{ef}), and the core contribution to the improved annihilation rates (Γ_{ef}^{co}) for isolated and solvated glycine (Gly). The correlation coefficient for the linear regressions of Γ_{HF} and PBE_{HF} is also shown. The results are indicated as ensemble averages and standard error of the average, except for isolated amino acid.

Species	$\Gamma_{HF}(10^{-2}ns^{-1})$	$\Gamma_{HF}^{co}(10^{-2}ns^{-1})$	$\Gamma_{ef}(10^{-2}ns^{-1})$	$\Gamma_{ef}^{co}(10^{-2}ns^{-1})$	R^2
Ala	10.22	0.08	35.50	0.05	
$Ala(H_2O)_1$	9.93 ± 0.22	0.40 ± 0.04	39.71 ± 0.96	0.50 ± 0.05	0.61
$Ala(H_2O)_2$	9.84 ± 0.31	0.65 ± 0.04	38.26 ± 1.28	0.82 ± 0.05	0.69
$Ala(H_2O)_3$	9.78 ± 0.34	0.82 ± 0.04	37.30 ± 1.37	1.03 ± 0.05	0.68
$Ala(H_2O)_4$	9.82 ± 0.35	0.97 ± 0.05	36.86 ± 1.35	1.21 ± 0.06	0.45
$Ala(H_2O)_5$	9.41 ± 0.37	1.02 ± 0.05	34.94 ± 1.38	1.28 ± 0.06	0.53
$Ala(H_2O)_6$	9.00 ± 0.35	1.06 ± 0.04	33.05 ± 1.33	1.33 ± 0.05	0.59
$Ala(H_2O)_7$	8.92 ± 0.39	1.11 ± 0.05	32.53 ± 1.47	1.39 ± 0.06	0.59
$Ala(H_2O)_{14}$	9.77 ± 0.67	1.44 ± 0.10	34.82 ± 2.40	1.81 ± 0.12	0.60
$Ala(H_2O)_{30}$	8.16 ± 0.63	1.45 ± 0.10	28.69 ± 2.26	1.81 ± 0.13	0.69

Table S16: Hartree-Fock annihilation rates (Γ_{HF}), contribution from core orbitals to the Hartree-Fock rates (Γ_{HF}^{co}), improved estimates obtained with enhancement factors (Γ_{ef}), and the core contribution to the improved annihilation rates (Γ_{ef}^{co}) for isolated and solvated alanine (Ala). The correlation coefficient for the linear regressions of Γ_{HF} and PBE_{HF} is also shown. The results are indicated as ensemble averages and standard error of the average, except for isolated amino acid.

Species	$\Gamma_{HF}(10^{-2}ns^{-1})$	$\Gamma_{HF}^{co}(10^{-2}ns^{-1})$	$\Gamma_{ef}(10^{-2}ns^{-1})$	$\Gamma_{ef}^{co}(10^{-2}ns^{-1})$	R^2
Pro	11.54	0.09	40.898	0.06	
$Pro(H_2O)_1$	10.75 ± 0.20	0.43 ± 0.04	43.36 ± 0.83	0.54 ± 0.05	0.63
$Pro(H_2O)_2$	10.00 ± 0.27	0.62 ± 0.04	39.52 ± 1.13	0.77 ± 0.05	0.72
$Pro(H_2O)_3$	9.66 ± 0.32	0.78 ± 0.04	37.46 ± 1.31	0.98 ± 0.05	0.79
$Pro(H_2O)_4$	9.39 ± 0.35	0.89 ± 0.04	35.96 ± 1.42	1.11 ± 0.05	0.80
$Pro(H_2O)_5$	9.11 ± 0.35	0.95 ± 0.04	34.44 ± 1.40	1.19 ± 0.05	0.79
$Pro(H_2O)_6$	9.13 ± 0.34	1.00 ± 0.04	34.24 ± 1.35	1.25 ± 0.05	0.75
$Pro(H_2O)_7$	9.29 ± 0.37	1.07 ± 0.04	34.69 ± 1.45	1.34 ± 0.05	0.76
$Pro(H_2O)_{14}$	10.06 ± 0.58	1.37 ± 0.07	36.93 ± 2.24	1.72 ± 0.09	0.71
$Pro(H_2O)_{30}$	9.16 ± 0.62	1.58 ± 0.10	32.59 ± 2.30	1.98 ± 0.12	0.68

Table S17: Hartree-Fock annihilation rates (Γ_{HF}), contribution from core orbitals to the Hartree-Fock rates (Γ_{HF}^{co}), improved estimates obtained with enhancement factors (Γ_{ef}), and the core contribution to the improved annihilation rates (Γ_{ef}^{co}) for isolated and solvated proline (Pro). The correlation coefficient for the linear regressions of Γ_{HF} and PBE_{HF} is also shown. The results are indicated as ensemble averages and standard error of the average, except for isolated amino acid.

7 Electrostatic potential and contact density

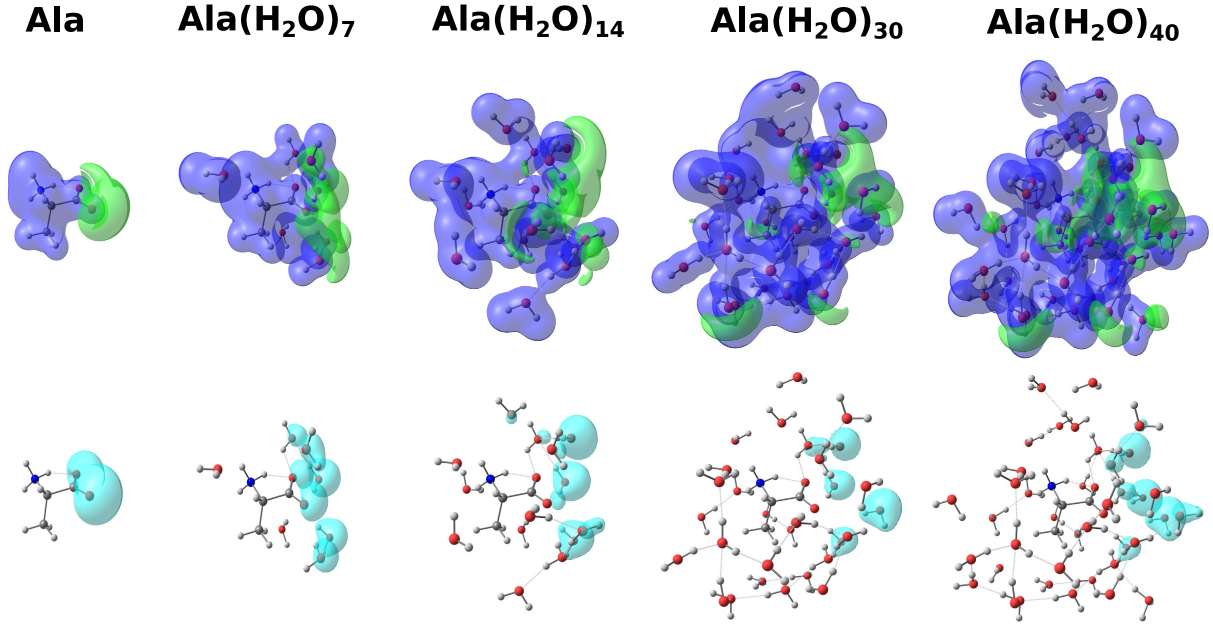

Figure S8: Electrostatic potential and contact density for alanine isolated and solvated. The electrostatic potential is represented by blue (positive) and green (negative) isosurfaces with the isovalue 0.1, while contact density by cyan isosurface with the isovalue 8×10^{-6}.

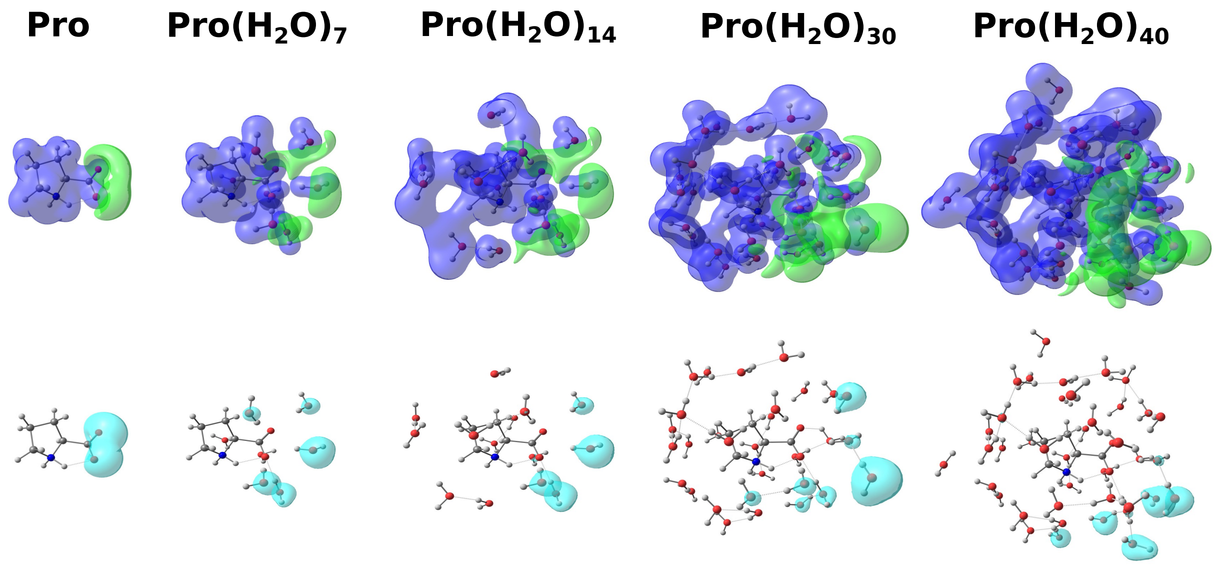

Figure S9: Electrostatic potential and contact density for proline isolated and solvated. The electrostatic potential is represented by blue (positive) and green (negative) isosurfaces with the isovalue 0.1, while contact density by cyan isosurface with the isovalue 8×10^{-6}.
